# HLA-B*15:01-positive severe COVID-19 patients lack CD8^+^ T cell pools with highly expanded public clonotypes

**DOI:** 10.1073/pnas.2503145122

**Published:** 2025-09-02

**Authors:** Louise C. Rowntree, Lilith F. Allen, Ruth R. Hagen, Hayley A. McQuilten, Ahmed A. Quadeer, Priyanka Chaurasia, Prathanporn Kaewpreedee, Kelly W. K. Lee, Carolyn A. Cohen, Jan Petersen, Dene R. Littler, Jennifer R. Habel, Wuji Zhang, Samuel M. S. Cheng, Ken Ka Pang Chan, Janette S. Y. Kwok, Kathy S. M. Leung, Joseph T. Wu, Cheuk-Kwong Lee, Jane Davies, Pia S. Pannaraj, E. Kaity Allen, Paul G. Thomas, Shidan Tosif, Nigel W. Crawford, Martha Lappas, Irani Thevarajan, Sharon R. Lewin, Stephen J. Kent, Jennifer A. Juno, Katherine A. Bond, Deborah A. Williamson, Natasha E. Holmes, Olivia C. Smibert, Claire L. Gordon, Jason A. Trubiano, Tom C. Kotsimbos, Allen C. Cheng, Claudia Efstathiou, Lance Turtle, Ryan S. Thwaites, Christopher E. Brightling, PHOSP-COVID Collaborative Group, Jamie Rossjohn, Matthew R. McKay, Jinmin Tian, William Jun Liu, George Fu Gao, Jianqing Xu, Kyuto Sonehara, Ken J. Ishii, Ho Namkoong, Yukinori Okada, Malik Peiris, David S. C. Hui, Leo L. M. Poon, Peter C. Doherty, Thi H. O. Nguyen, Sophie A. Valkenburg, Katherine Kedzierska

**Affiliations:** ^a^Department of Microbiology and Immunology, University of Melbourne, at the Peter Doherty Institute for Infection and Immunity, Melbourne, VIC 3000, Australia; ^b^Department of Electronic and Computer Engineering, School of Engineering, Hong Kong University of Science and Technology, Hong Kong Special Administrative Region, Hong Kong, China; ^c^Department of Electrical and Electronic Engineering, University of Melbourne, Melbourne, VIC 3010, Australia; ^d^Infection and Immunity Program and Department of Biochemistry and Molecular Biology, Biomedicine Discovery Institute, Monash University, Clayton, VIC 3800, Australia; ^e^HKU-Pasteur Research Pole, School of Public Health, The University of Hong Kong, Hong Kong Special Administrative Region, Hong Kong, China; ^f^Division of Public Health Laboratory Sciences, School of Public Health, The University of Hong Kong, Hong Kong Special Administrative Region, Hong Kong, China; ^g^Department of Medicine and Therapeutics, Prince of Wales Hospital, The Chinese University of Hong Kong, Hong Kong Special Administrative Region, Hong Kong, China; ^h^Li Ka Shing Institute of Health Sciences, Faculty of Medicine, The Chinese University of Hong Kong, Hong Kong Special Administrative Region, Hong Kong, China; ^i^Division of Transplantation and Immunogenetics, Department of Pathology, Queen Mary Hospital, Hong Kong Special Administrative Region, Hong Kong, China; ^j^The Hong Kong Jockey Club Global Health Institute, Hong Kong Special Administrative Region, Hong Kong, China; ^k^WHO Collaborating Centre for Infectious Disease Epidemiology and Control, School of Public Health, Li Ka Shing Faculty of Medicine, The University of Hong Kong, Hong Kong Special Administrative Region, Hong Kong, China; ^l^Laboratory of Data Discovery for Health, Hong Kong Science Park, Hong Kong Special Administrative Region, Hong Kong, China; ^m^The University of Hong Kong - Shenzhen Hospital, Shenzhen 518009, China; ^n^Menzies School of Health Research, Darwin, NT 0810, Australia; ^o^Division of Infectious Diseases, Children’s Hospital Los Angeles, Los Angeles, CA 90027; ^p^Department of Pediatrics, Molecular Microbiology and Immunology, Keck School of Medicine, The University of California, Los Angeles, CA 90033; ^q^Department of Immunology, St. Jude Children’s Research Hospital, Memphis, TN 38105; ^r^Infection and Immunity, Murdoch Children’s Research Institute, Melbourne, VIC 3052, Australia; ^s^Department of Paediatrics, University of Melbourne, Melbourne, VIC 3010, Australia; ^t^Department of General Medicine, Royal Children’s Hospital Melbourne, Melbourne, VIC 3052, Australia; ^u^Royal Children’s Hospital Melbourne, Immunisation Service, Melbourne, VIC 3052, Australia; ^v^Department of Obstetrics, Gynaecology and Newborn Health, University of Melbourne, Melbourne, VIC 3010, Australia; ^w^Department of Infectious Diseases, University of Melbourne, at the Peter Doherty Institute for Infection and Immunity, Melbourne, VIC 3000, Australia; ^x^Victorian Infectious Diseases Service, Royal Melbourne Hospital, at the Peter Doherty Institute for Infection and Immunity, Melbourne, VIC 3000, Australia; ^y^Department of Infectious Diseases, Alfred Hospital and Monash University, Melbourne, VIC 3004, Australia; ^z^ARC Centre of Excellence in Convergent Bio-Nano Science and Technology, University of Melbourne, Melbourne, VIC 3010, Australia; ^aa^Melbourne Sexual Health Centre, Infectious Diseases Department, Alfred Health, Central Clinical School, Monash University, Melbourne, VIC 3004, Australia; ^bb^Victorian Infectious Diseases Reference Laboratory, at the Peter Doherty Institute for Infection and Immunity, Melbourne, VIC 3000, Australia; ^cc^Department of Microbiology, Royal Melbourne Hospital, Melbourne, VIC 3050, Australia; ^dd^School of Medicine, University of St Andrews, Scotland KY16 9TF, United Kingdom; ^ee^Department of Infectious Diseases and Immunology, Austin Health, Heidelberg, VIC 3084, Australia; ^ff^Data Analytics Research and Evaluation Centre, Austin Health and University of Melbourne, Heidelberg, VIC 3084, Australia; ^gg^Department of Infectious Diseases, Centre for Antibiotic Allergy and Research, Austin Health, Heidelberg, VIC 3084, Australia; ^hh^Department of Infectious Diseases, Peter MacCallum Cancer Centre, Melbourne, VIC 3000, Australia; ^ii^National Centre for Infections in Cancer, Peter McCallum Cancer Centre, Melbourne, VIC 3000, Australia; ^jj^Department of Respiratory Medicine, The Alfred Hospital, Melbourne, VIC 3004, Australia; ^kk^Department of Medicine, Central Clinical School, The Alfred Hospital, Monash University, Melbourne, VIC 3004, Australia; ^ll^School of Public Health and Preventive Medicine, Monash University, Clayton, VIC 3004, Australia; ^mm^Monash Infectious Diseases, Monash Health and School of Clinical Sciences, Monash University, Clayton, VIC 3168, Australia; ^nn^National Heart and Lung Institute, Imperial College London, London SW3 6LY, United Kingdom; ^oo^Institute of Infection, Ecological and Veterinary Sciences, University of Liverpool & Liverpool University Hospital National Health Service Foundation Trust, Wirral CH64 7TE, United Kingdom; ^pp^Institute for Lung Health, Leicester NIHR BRC, University of Leicester, Leicester LE1 7RH, United Kingdom; ^qq^Institute of Infection and Immunity, Cardiff University School of Medicine, Cardiff CF14 4XN, United Kingdom; ^rr^National Institute for Viral Disease Control and Prevention, Chinese Center for Disease Control and Prevention, Beijing 102206, China; ^ss^Shanghai Public Health Clinical Centre and Institutes of Biomedical Sciences, Key Laboratory of Medical Molecular Virology of Ministry of Education/Health, Shanghai Medical College, Fudan University, Shanghai 201508, China; ^tt^Department of Statistical Genetics, Osaka University Graduate School of Medicine, Suita 565-0871, Japan; ^uu^Department of Genome Informatics, Graduate School of Medicine, University of Tokyo, Tokyo 113-8654, Japan; ^vv^Laboratory of Adjuvant Innovation, Center for Vaccine and Adjuvant Research, National Institutes of Biomedical Innovation, Health and Nutrition, Saito Asagi, Ibaraki 567-0085, Osaka, Japan; ^ww^Division of Vaccine Science, Department of Microbiology and Immunology, The Institute of Medical Science, The University of Tokyo, Minato-ku, Tokyo 108-8639, Japan; ^xx^Department of Immunopathology, Immunology Frontier Research Center, Osaka University, Suita 565-0871, Japan; ^yy^Department of Infectious Diseases, Keio University School of Medicine, Tokyo 160-8582, Japan; ^zz^Laboratory for Systems Genetics, RIKEN Center for Integrative Medical Sciences, Yokohama 230-0045, Japan; ^aaa^Premium Research Institute for Human Metaverse Medicine, Osaka University, Suita 565-0871, Japan; ^bbb^Centre for Immunology and Infection, Hong Kong Science and Technology Park, New Territories, Hong Kong Special Administrative Region, Hong Kong, China; ^ccc^Institute for Vaccine Research and Development, Hokkaido University, Sapporo 001-0021, Japan

**Keywords:** CD8+ T cells, T cell receptors, HLA-B*15:01, COVID-19, severe disease

## Abstract

Understanding host factors driving asymptomatic versus severe disease outcomes is of key importance if we are to control emerging and re-emerging viral infections. HLA-B*15:01 has been associated with asymptomatic SARS-CoV-2 infection in nonhospitalized individuals of European ancestry, with protective immunity attributed to preexisting cross-reactive CD8^+^ T-cells directed against HLA-B*15:01-restricted Spike-derived S_919-927_ peptide (B15/S_919_^+^CD8^+^ T-cells). However, fundamental questions remained on the abundance and clonotypic nature of CD8^+^ T-cell responses in HLA-B*15:01-positive patients who succumbed to life-threatening COVID-19. Here, we analyzed B15/S_919_^+^CD8^+^ T-cell responses in COVID-19 patients from independent HLA-typed COVID-19 patient cohorts across three continents, Australia, Asia and Europe. We assessed B15/S_919_^+^CD8^+^ T-cells in COVID-19 patients across disease outcomes ranging from asymptomatic to hospitalized critical illness. We found that severe/critical COVID-19 patients mounted B15/S_919_^+^CD8^+^ T-cell responses lacking a highly expanded key public B15/S_919_^+^CD8^+^ T-cell receptor (TCR; TRAV9-2/TRBV7-2) which recurred across multiple individuals in COVID-19 patients with a mild disease. Instead, B15/S_919_^+^CD8^+^ T-cell responses in life-threatening disease had a prevalence of an alternate TCR clonotypic motif (TRAV38-2/DV8/TRBV20-1), potentially contributing, at least in part, to why B15/S_919_^+^CD8^+^ T-cells in severe COVID-19 patients were less protective. Interestingly, the frequency, memory phenotype, and activation profiles of circulating B15/S_919_^+^CD8^+^ T-cells did not differ across disease severity. Moreover, B15/S_919_^+^CD8^+^ T-cells were better maintained into convalescence compared to other SARS-CoV-2-specificities. Our study thus provides evidence on the differential nature of the TCR clonal repertoire in 22.37% of HLA-B*15:01-positive COVID-19 patients who developed severe or critical disease in our cohorts, comparing to HLA-B*15:01-expressing individuals with mild COVID-19.

## Significance

Understanding factors driving asymptomatic versus severe disease is of key importance if we are to control emerging and re-emerging viral infections. As preexisting CD8^+^ T-cell responses have been associated with asymptomatic COVID-19 in individuals expressing HLA-B*15:01, comparing to mild disease, we defined cross-reactive CD8^+^ T-cells responses directed at the HLA-B*15:01/S_919_ epitope in COVID-19 patients across disease outcomes ranging from asymptomatic to critical illness. We found that severe COVID-19 patients had an enrichment of an alternate T-cell receptor (TCR) motif compared to the key public motif expanded in milder patients. Our study provides evidence on differential nature of TCR clonal repertoire in 22.37% of HLA-B*15:01-positive COVID-19 patients who developed severe/critical disease in our cohorts, comparing to HLA-B*15:01-expressing individuals with mild COVID-19.

Prior to the vaccination rollout, the spectrum of disease severity associated with severe acute respiratory syndrome coronavirus 2 (SARS-CoV-2) infection ranged from 14% and 50% for potentially asymptomatic cases (1) to 13% needing hospitalization and 3.2% fatal cases (2). While robust immunity elicited via COVID-19 vaccination and prior SARS-CoV-2 infections reduced SARS-CoV-2-associated mortality and morbidity, it is still of key importance to understand host factors underpinning asymptomatic versus severe disease outcomes if we are to prevent life-threatening disease to emerging and re-emerging viral infections, especially in vulnerable populations.

Outside of impaired type-I interferon immunity, which confers elevated susceptibility in at least 15% of severe COVID-19 cases (3), it is unclear why some previously healthy individuals develop severe disease while others remain asymptomatic. HLA polymorphisms have been linked to various disease outcomes, including both protection and severity associated with viral infections (4–8). Potential associations between HLA alleles and COVID-19 severity were investigated by a number of studies; the largest of which found no association between 66 of the most common HLA loci and SARS-CoV-2 infection or hospitalization (n = 6,413 COVID-19-positive Israeli individuals) (9). Conversely, a study found HLA-B*15:01 associated with asymptomatic SARS-CoV-2 in a mildly infected cohort of European ancestry (n = 1,428) (4). Cross-reactivity between a HLA-B*15:01-restricted SARS-CoV-2 Spike-derived CD8^+^ T cell epitope B15/S_919-927_ (NQKLIANQF) and a seasonal human coronavirus (hCoV) epitope B15/S_1012-1020_ (NQKLIANAF from OC43-hCoV and HKU1-hCoV) (5) led this study to hypothesize the genetic association might be due to preexisting immunity. T cells from prepandemic samples were reactive to both SARS-CoV-2 B15/S_919_ and hCoV B15/S_1012_ epitopes, with most responding CD8^+^ T cells having a memory phenotype. Additionally, T cells from both prepandemic and COVID-19 vaccinated individuals shared common TCR features, with presence of public (identical TCRs recurred in multiple individuals) and cross-reactive (recognizing multiple peptide variants) TCRs (4, 5), which is of importance as TCR diversity and clonal signatures can affect T cell immunodominance, functionality, and protection (10–12). Structural similarity of the HLA-B*15:01 molecule presenting both peptide variants, which differ by only one amino acid at position 8, suggested a molecular basis for T cell cross-reactivity (4).

Robust CD8^+^ T cell responses directed toward another immunodominant SARS-CoV-2 epitope (B7/N_105-113_) have also been strongly associated with reduced viral load and mild COVID-19 (13). Protective B7/N_105_^+^CD8^+^ T cell responses in mild COVID-19 patients displayed higher functional avidity as well as optimal effector and antiviral CD8^+^ T cell functions, in contrast to suboptimal CD8^+^ T cell responses in severely ill patients. Direct ex vivo evidence demonstrated recruitment of naïve B7/N_105_^+^CD8^+^ T cell pools rather than preexisting cross-reactive memory CD8^+^ T cell populations (13, 14). In contrast to the biased and public TCR repertoire of B15/S_919_, the B7/N_105_-specific TCR repertoire was highly diverse (14). Peng et al. found the B7/N_105_-specific TCR repertoire in mildly infected patients shared higher similarity with the prepandemic TCR repertoire than that from severe COVID-19 patients, suggesting that protective effects resulted from early, preferential expansion of naïve high-frequency, high-functional avidity B7/N_105_-specific clonotypes (13). However, while B7/N_105_-specific CD8^+^ T cell responses were associated with protection against severe COVID-19, HLA-B*07:02 allele expression was not associated with disease outcome.

As HLA-B*15:01 was associated with asymptomatic SARS-CoV-2 infection in comparison to mild COVID-19 in nonhospitalized individuals of European-ancestry (4), we sought to answer fundamental questions on the abundance and clonotypic nature of CD8^+^ T cell responses in HLA-B*15:01-positive COVID-19 patients who succumbed to life-threatening COVID-19. Our ex vivo approach analyzed B15/S_919_^+^CD8^+^ T cell responses in unvaccinated COVID-19 patients from independent HLA-typed COVID-19 cohorts across 3 continents, Australia, Asia and Europe. We assessed HLA-B*15:01-expressing COVID-19 patients across the disease severity spectrum, from asymptomatic and mild infections, to hospitalized moderate and severe/critical patients, as well as prepandemic unexposed individuals. We found that patients across disease severities had comparable levels of circulating B15/S_919_^+^CD8^+^ T cells, however severe/critical patients had reduced expansion of the key public B15/S_919_^+^CD8^+^ TCR (TRAV9-2/TRBV7-2) but enrichment of an alternate TCR motif (TRAV38-2/DV8/TRBV20-1). Additionally, our analysis of four independent cohorts of Asian ancestry (n = 4,930), found no significant associations between HLA alleles and asymptomatic SARS-CoV-2 infection, suggesting the HLA-B*15:01 association with asymptomatic infection is not a global phenomenon and may be restricted by ancestry.

## Results

### B15/S_919_^+^CD8^+^ T Cells Maintained across Time and Disease Severity.

**Fig. 1.**
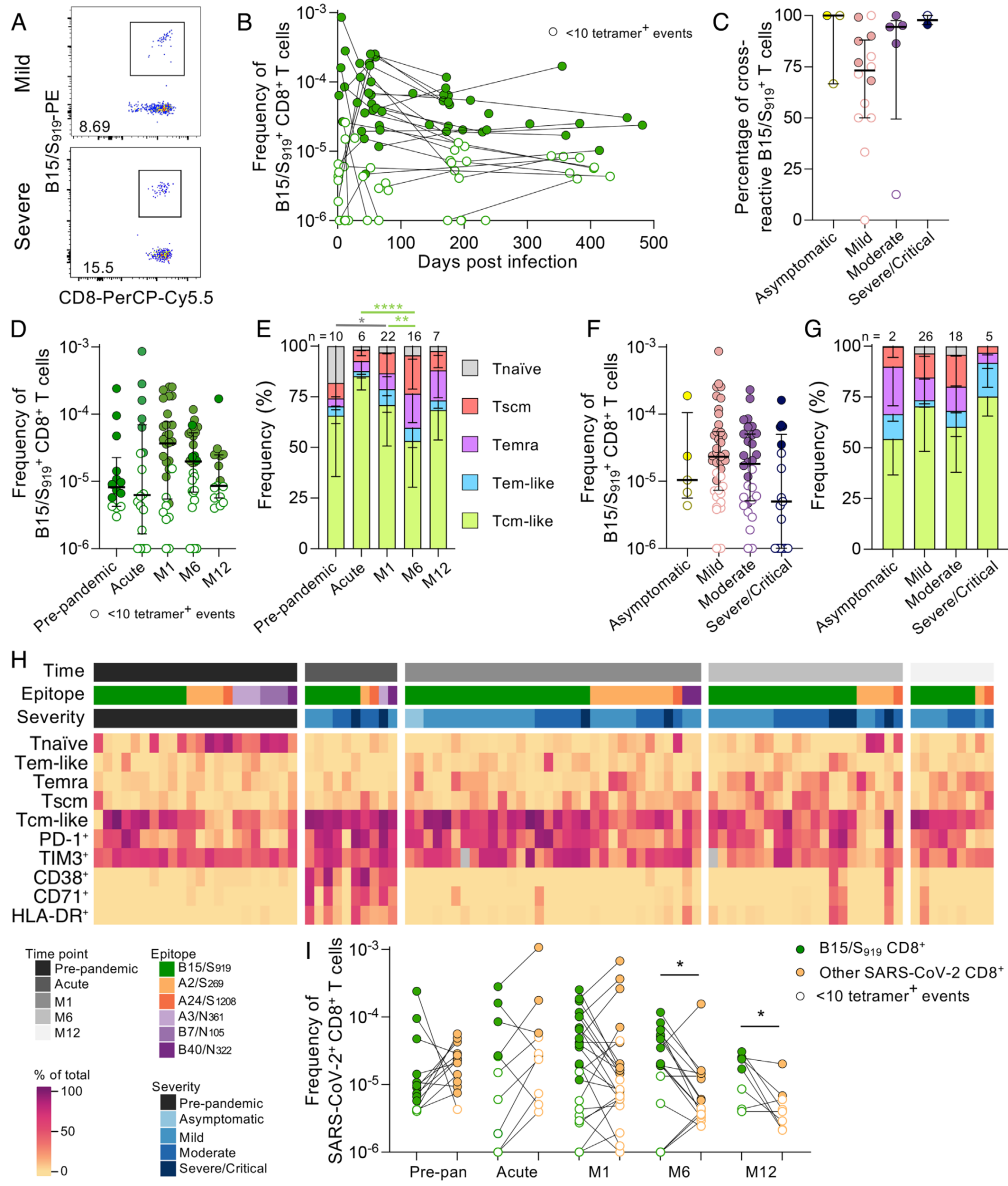
B15/S_919_^+^CD8^+^ T cell frequency and phenotype are maintained across time and disease severity. (*A*) Representative flow cytometry plots of enriched B15/S_919_-specific tetramer^+^ CD8^+^ T cells. (*B*) B15/S_919_^+^CD8^+^ T cell frequencies across time following primary SARS-CoV-2 infection. (*C*) Percentage of B15/S_919_^+^CD8^+^ T cells cross-reactive with hCoV B15/S_1012_ across disease severity groups, median with interquartile range (IQR). (*D*) Frequency of B15/S_919_^+^CD8^+^ T cells prepandemic and across time post infection, median with IQR. (*E*) Memory phenotype profiles across time for B15/S_919_^+^CD8^+^ T cells, mean with SD. (*F*) Frequency of B15/S_919_^+^CD8^+^ T cells across disease severity groups, median with IQR. (*G*) Phenotype profiles across disease severity groups for B15/S_919_^+^CD8^+^ T cells, mean with SD. (*H*) Heatmap showing activation (CD71, CD38, HLA-DR, PD-1, and TIM-3) and memory phenotype of SARS-CoV-2 epitope-specific CD8^+^ T cells. (*I*) Matched B15/S_919_^+^ and other SARS-CoV-2^+^CD8^+^ T cell frequencies within individuals across time. Statistical significance determined by (*C*, *D*, and *F*) Dunn’s multiple comparisons test, (*E* and *G*) Tukey’s multiple comparisons test and (*I*) Wilcoxon matched-pairs signed rank test. *P ≤ 0.05, **P ≤ 0.01, ***P ≤ 0.001, ****P ≤ 0.0001. Frequency of tetramer^+^CD8^+^ T cells are shifted by 10^−6^ (i.e., no detected tetramer^+^ events displayed as 10^−6^) to allow for visibility on a logarithmic *y* axis. Any samples with <10 tetramer^+^ events are shown as open symbols and only samples with 10 or more tetramer^+^ events are included in the phenotypic analysis (*E*, *G*, and *H*). For samples run in duplicate, averaged tetramer^+^ T cell frequencies are plotted.

To determine B15/S_919_-specific CD8^+^ T cell responses in COVID-19 patients across disease severities (*SI Appendix*, Fig. S1*A*), we analyzed cellular responses of 45 unvaccinated HLA-B*15:01-expressing individuals, either asymptomatic (n = 3), symptomatic but recovering at home (n = 14 mild) or hospitalized (n = 15 moderate, n = 13 severe/critical), with prepandemic/preinfection PBMC samples (n = 14) for comparison. HLA/peptide (HLA/p) tetramers combined with tetramer-associated magnetic enrichment were used to directly assess ex vivo CD8^+^ T cell responses against the B*15:01/S_919_ epitope across 12 mo following primary SARS-CoV-2 infection (Fig. 1 *A* and *B*). Cross-reactivity of B15/S_919_-specific CD8^+^ T cells with hCoV B15/S_1012_ was confirmed in a subset of participants (n = 13), with 74.57% of B15/S_919_-specific CD8^+^ T cells costaining with the B15/S_1012_ tetramer and no difference in cross-reactivity across disease severity groups (Fig. 1*C*). In accordance with the cross-reactive nature of the B15/S_919_-specific CD8^+^ T cell response, we observed comparable ex vivo frequencies of B15/S_919_^+^CD8^+^ T cells between prepandemic samples and samples from acute or recovered SARS-CoV-2 infection across time (Fig. 1*D*). While the B15/S_919_^+^CD8^+^ T cells had predominantly CD45RA^−^CD27^+^ central memory-like phenotype in both prepandemic and post-SARS-CoV-2 infection samples, infection still resulted in a decrease in CD45RA^+^CD27^+^CD95^-^ naïve-like B15/S_919_^+^CD8^+^ T cells by 1 mo (Fig. 1*E*). Interestingly, the proportion of central memory T cells decreased at 6 mo postinfection compared to acute or 1-mo postinfection (Fig. 1*E*). Within SARS-CoV-2-infected participants, the frequency of circulating B15/S_919_^+^CD8^+^ T cells was decreased in severe/critical disease compared to mild infection (Mann–Whitney; *P* = 0.0282) (Fig. 1*F*). However, when all severity groups were compared against each other, there was no difference in B15/S_919_^+^CD8^+^ T cell frequency or memory phenotype between groups (Dunn’s multiple comparisons test, Fig. 1 *F* and *G*). Finally, during acute infection, B15/S_919_^+^CD8^+^ T cells transiently expressed prototypical activation markers (CD71, CD38, HLA-DR), while PD-1 and TIM-3, which are typically associated with previous antigen-experience (15), were maintained into convalescence (Fig. 1*H*).

Other immunodominant SARS-CoV-2 CD8^+^ (A2/S_269_, A3/N_361_, A24/S_1208_, B7/N_105_, and B40/N_322_) T cell responses were also examined based on HLA availability (14, 16–19). Interestingly, when the frequency of paired B15/S_919_ CD8^+^ T cells and other SARS-CoV-2^+^CD8^+^ T cells were analyzed, the frequency of other SARS-CoV-2 tetramer^+^CD8^+^ T cells was lower than the B15/S_919_-specific CD8^+^ T cells at 6-mo (*P* = 0.0302) and 12-mo (*P* = 0.0312) postinfection, indicating better maintenance of the B15/S_919_^+^CD8^+^ T cells into long-term memory (Fig. 1*I*). A similar pattern was observed in other SARS-CoV-2 CD8^+^ T cells, which decreased by 6- and 12-mo postinfection compared to prepandemic (M12: *P* = 0.0208) or acute infection (M6: *P* = 0.0495; M12: *P* = 0.0160) (*SI Appendix*, Fig. S1*B*). These SARS-CoV-2 CD8^+^ T cell specificities shifted from a prototypical prepandemic naïve phenotype to a more central memory phenotype with acute COVID-19 (*SI Appendix*, Fig. S1*C*). Interestingly, we observed a larger decrease in the frequency of other SARS-CoV-2-specific CD8^+^ T cells with a central memory phenotype over time following infection compared to the B15/S_919_^+^CD8^+^ T cell population (Fig. 1*E*), suggesting better maintenance of the B15/S_919_-specificity. Frequency and memory phenotype for the other SARS-CoV-2^+^CD8^+^ T cell populations were relatively unchanged across disease severity groups (*SI Appendix*, Fig. S1 *D* and *E*). The severe/critical group had a higher frequency of central memory SARS-CoV-2^+^CD8^+^ T cells compared to mild or moderate disease, however this is likely a feature of time postinfection, with our cohort having fewer severe/critical samples >250 d postinfection. Finally, we analyzed a CD4^+^ restricted SARS-CoV-2 specificity (DPB4/S_167_) (18–20) and found the ex vivo frequency increased following infection and was maintained for 12 mo (M1: *P* = 0.0003; M6: *P* = 0.0029; M12: *P* = 0.0061) (*SI Appendix*, Fig. S1*F*). Meanwhile, the phenotype of DPB4/S_167_^+^CD4^+^ T cells was over 80% central memory during and following SARS-CoV-2 infection and was unaffected by disease severity (*SI Appendix*, Fig. S1 *G*–*I*). Similarly, IgG titers specific for the ancestral RBD were unaffected by time postinfection or disease severity (*SI Appendix*, Fig. S1 *J* and *K*).

Overall, our data suggest that preexisting cross-reactive memory pools of B15/S_919_^+^CD8^+^ T cells had no numerical advantage during acute SARS-CoV-2 infection, however they were better maintained to >12 mo post primary infection.

### Altered B15/S_919_-Specific TCR Repertoire in Severe/Critical COVID-19 Patients.

As public TCR features within B15/S_919_^+^CD8^+^ T cells were attributed to protective immunity in HLA-B*15:01-expressing individuals (4), we determined the B15/S_919_-specific TCR repertoire across disease severities using single-cell TCRαβ multiplex-nested RT-PCR after ex vivo tetramer-enrichment. A total of 717 B15/S_919_^+^CD8^+^ T cells (including 530 paired TCRαβ) from 63 samples, representing 48 individuals from prepandemic, asymptomatic, mild and hospitalized moderate, severe/critical disease were analyzed for their TCR clonotype composition and clonal expansions (Dataset S1).

**Fig. 2.**
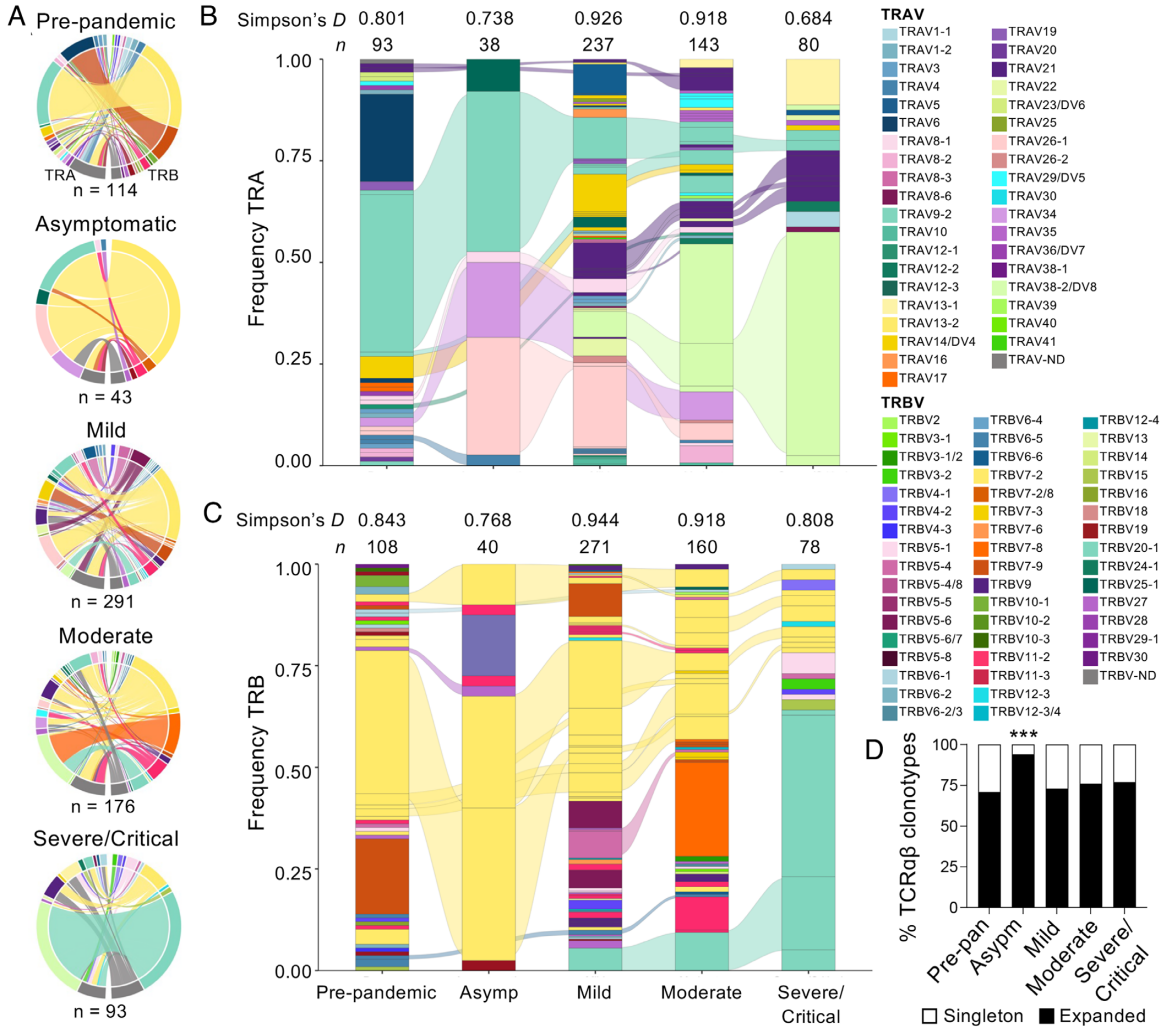
Altered B15/S_919_-specific TCR repertoire in severe/critical COVID-19 patients. (*A*) Circos plots depicting TRA and TRB gene usage for TCRαβ clonotypes specific to B15/S_919_. Left outer arch color indicates TRAV usage, right outer arch and segment color depicts TRBV usage (n = sequences). (*B* and *C*) Alluvial plots showing frequency of TRA (*B*) and TRB (*C*) gene usage in B15/S_919_-specific TCR repertoires (n = sequences). Connections between bars represent shared CDR3 usage between individuals of different COVID-19 severity. (*A*–*C*) Colors represent variable gene segment usage, while divisions represent TCRαβ clonotypes with the same CDR3 sequence. (*D*) Overall proportion of singleton and expanded clonotypes (≥2 clonotypes in one donor) from total TCRαβ sequences per disease group and prepandemic. Statistical significance determined by (*B* and *C*) Simpson’s Diversity Index and (*D*) Fisher’s exact test for comparison between disease groups. *P ≤ 0.05, **P ≤ 0.01, ***P ≤ 0.001, ****P ≤ 0.0001.

In line with previous reports describing TCRαβ repertoires from prepandemic and COVID-19 vaccinated individuals within B15/S_919_^+^CD8^+^ T cells (4, 5), the ex vivo TCR repertoire from prepandemic samples was strongly biased toward clonotypes expressing TRAV9-2 paired with TRBV7-2 (33.3%), which we refer to as one of the key public TCR pairings for B15/S_919_^+^CD8^+^ T cells (Fig. 2*A* and Dataset S1). We also found a TRAV6/TRBV7-9 pairing that was highly expanded in one individual, contributing to 16.7% of the overall prepandemic repertoire. In SARS-CoV-2-infected individuals, TRAV9-2/TRBV7-2 clonotypes were observed across disease severity groups, however the abundance of this key public TCR pairing decreased, with increasing disease severity (asymptomatic 32.56%, mild 9.97%, moderate 7.39%, severe/critical 3.26%) (Fig. 2*A*).

Variable gene usage and CDR3α and CDR3β loops were shared across prepandemic and COVID-19 severity groups (Fig. 2 *B* and *C*). TRBV7-2 accounted for 38.75 to 85.00% of the TCRβ repertoire in prepandemic, asymptomatic, mild and moderate disease groups; however, only 16.88% in the severe/critical TCR repertoire (Fig. 2*C*). Instead, the severe/critical TCRβ repertoire was dominated by TRBV20-1 (63.64%; 4/6 severe/critical individuals), this variable gene was observed at lower frequencies in the mild (5.54%) and moderate (9.38%) groups. Previously observed bias for TRAV21 in unexposed and vaccinated individuals (4, 5) was surprisingly rare or absent in our prepandemic (2/93 alpha sequences from 12 individuals, representing 2.15%) and asymptomatic (0/38 alpha sequences from two individuals) participants, but represented 10.55%, 11.89%, and 12.66% in mild, moderate, and severe/critical disease TCR repertoires, respectively (Fig. 2*B*).

Across the dataset, 62 out of 193 unique clonotypes (from 530 paired sequences) were expanded, with 32 out of 44 donors having at least one expanded clone (Dataset S1). Interestingly, the asymptomatic TCR repertoire comprised the highest proportion of expanded TCRs (94.29%) compared to prepandemic and other severity groups (70.93 to 76.92%, *P* ≤ 0.0001) (Fig. 2*D*), suggesting that asymptomatic SARS-CoV-2 response may be driven by clonal expansions, though it is worth noting the asymptomatic TCR repertoire contained the lowest number of TCR sequences (n = 35).

Twelve TCRαβ clonotypes were identified in two or more individuals (Dataset S1). One prominent clonotype previously described in prepandemic and vaccinated individuals (4, 5) and representing one of the key public TCR pairings, TRAV9-2_TRAJ27_CALSDSNAGKSTF/TRBV7-2_TRBJ2-1_CASSLASESYNEQFF, was highly expanded in two prepandemic individuals (79.17% and 89.47% of individual’s TCR repertoire), one asymptomatic (14/27 sequences, 40.00%) and one mild patient (3/15 sequences, 16.67%), but was only found as a singleton in one severe patient (1/37 sequences, 2.08%). The other prominent TCRβ chain described in prepandemic, infected, and vaccinated individuals (4, 5), TRBV7-2_TRBJ1-2_CASSLEDTNYGYTF, bearing the same TRBV7-2 gene segment but different CDR3β-TRBJ gene to the above, was observed across 11 individuals from prepandemic, mild, and moderate groups. This TCRβ chain was paired with at least five different TCRα variable regions, including the public TRAV21_TRAJ40_CAVHTSGTYKYIF and related TCRα chains. A similar clonotype, TRAV21_TRAJ40_CAALTSGTYKYIF/TRBV7-2_TRBJ1-2_CASSLEDTIYGYTF), was observed across four individuals in our dataset (1 prepandemic, 1 mild, and 2 moderate individuals). The TCRαβ clonotype identified in the highest number of individuals, TRAV38-2/DV8_TRAJ43_CAYRFNNNDMRF/TRBV20-1_TRBJ1-1_CSATRDRGYTEAFF, was observed in one mild (37.5% of individual’s TCR repertoire), four moderate (6.90%, 9.13%, 18.18% and 40.00%), and two severe/critical (17.02% and 18.52%) patients. This TCR was previously observed in one prepandemic individual (4). Finally, TRAV34_TRAJ30_CGADIPNRDDKIIF/TRBV7-2_TRBJ2-3_CASRLAGQYSTDTQYF, was found as a singleton in two prepandemic individuals and expanded in one asymptomatic (21.10%) and one moderate patient (35.00%).

To determine whether decreased representation of the key TRAV9-2/TRBV7-2 public TCR pairing in severe/critical patients was unique to B15/S_919_^+^CD8^+^ T cells, we analyzed TCR sequences specific to non-cross-reactive A2/S_269_^+^CD8^+^ (sequences from 90 cells) and DPB4/S_167_^+^CD4^+^ T cells (sequences from 121 cells) with known TCR biases (Dataset S1) (18–21). For A2/S_269_^+^CD8^+^, the TRAV12-1_TRAJ43_CVVNXXDDMRF motif and TRBV7-9 bias were observed in mild, moderate, and severe/critical patients at similar frequencies (*SI Appendix*, Fig. S2*A*). Similarly, for DPB4/S_167_^+^CD4^+^, the highly prominent TRAV35_TRAJ42 CAXXNYGGSQGNLIF TCRα motif was observed in all disease severity groups (79.05% of TCRα) (*SI Appendix*, Fig. S2*B*).

### Severe/Critical Patients B15/S_919_-Specific TCR Repertoire Dominated by an Alternate Motif.

**Fig. 3.**
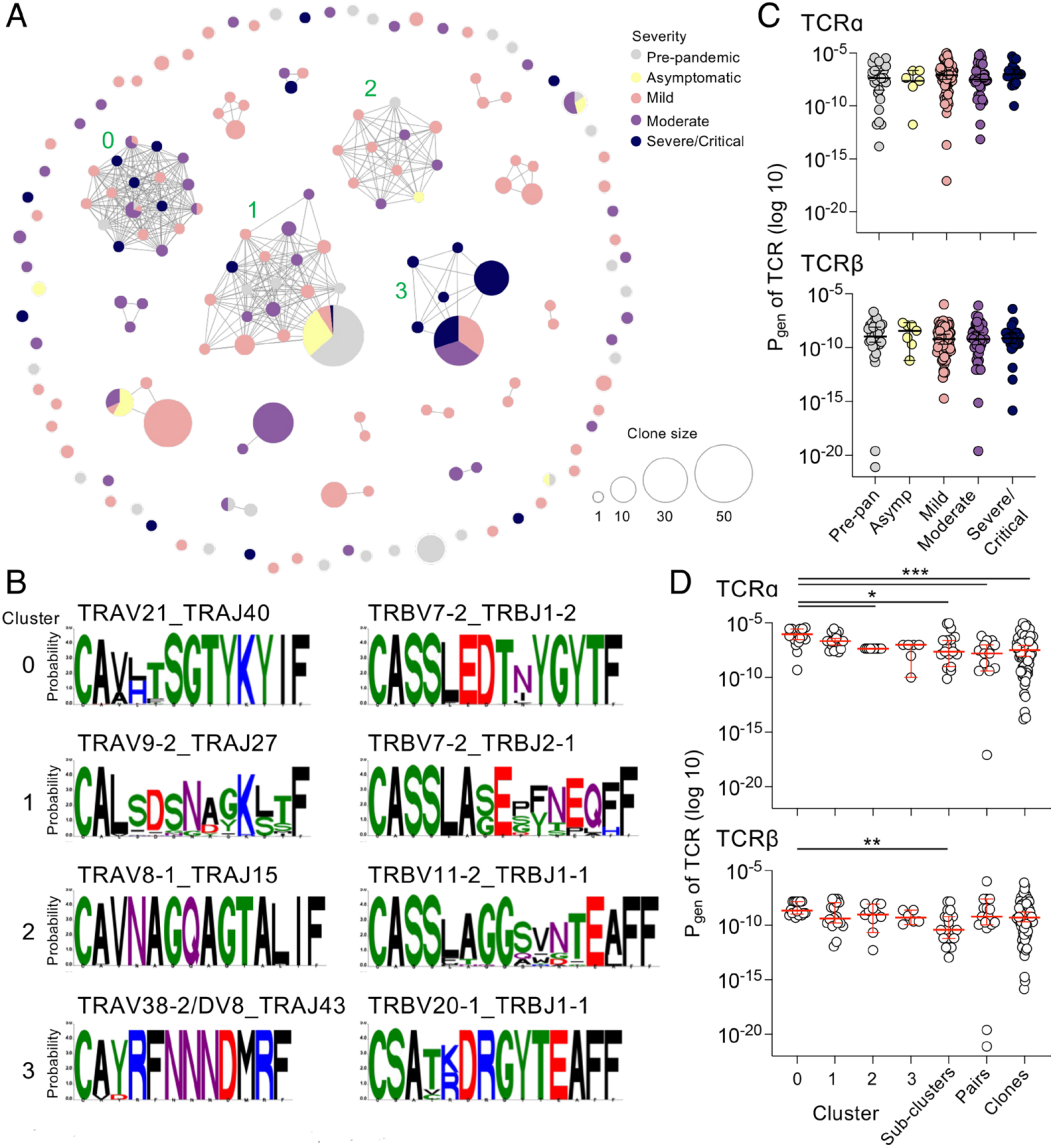
B15/S_919_-specific TCR repertoire in severe/critical patients dominated by an alternate motif. (*A*) Network analysis of paired B15/S_919_-specific TCRαβ clonotypes. Network nodes (circles) represent clonotypes of paired TCRαβ, and are connected by edges (lines) to other clonotypes that are ≤120 TCRdist units (22). Size of the nodes represents the size of the clonotypes. (*B*) Logo plots for networked clonotype clusters with ≥5 clonotypes detected in B15/S_919_-specific TCR repertoire. Cluster numbers indicated in (*A*). Sequence logo shows the probability of each amino acid residue at each CDR3 position. Colors represent amino acid chemistry: red, acid; blue, basic; black, hydrophobic; purple, neutral; green, polar. (*C* and *D*) Probability of generation (P_gen_; log10 transformed) for TCRα and TCRβ chains were generated with tcrdist3 (23) and plotted for (*C*) prepandemic and disease severity groups and (*D*) clusters. (*C*) Clonally expanded TCRs within an individual were reduced to a single data point for this analysis. (*D*) Subclusters represent clusters consisting of 3 or 4 clonotypes, pairs represent clusters consisting of two clonotypes and clones are singleton TCRs; clonally expanded TCRs were reduced to a single data point for this analysis. Plots represent the median with 95% CI. Statistical significance determined by (*C* and *D*) Dunn’s multiple comparisons test. *P ≤ 0.05, **P ≤ 0.01, ***P ≤ 0.001, ****P ≤ 0.0001.

To visualize sequence similarity within the B15/S_919_-specific TCR repertoire, we constructed a similarity network of paired TCRαβ sequences (Fig. 3*A*). Overall, we found 18 clusters within our B15/S_919_-specific TCR repertoire containing two or more clonotypes (Fig. 3*A*). Motifs were generated for four clusters containing ≥5 clonotypes using TCRdist3 (22) (Fig. 3*B*). Cluster 0 contained minimal clonal expansions with mainly single clonotypes identified from within prepandemic and all disease severity groups expect for asymptomatic. Cluster 1 contained clonotypes from prepandemic samples and all four disease severity groups, with large clonal expansions (>10 clones) from predominantly prepandemic, asymptomatic, and mild individuals. Cluster 0 and 1 represent previously published public TCR motifs TRAV21/TRBV7-2 and TRAV9-2/TRBV7-2, respectively (4, 5). Representation of the severe/critical TCR repertoire was limited in these highly abundant clusters. Cluster 2 contained a TRAV8-1/TRBV11-2 motif, where clonotypes bearing this motif were identified predominantly in mild patients and were generally single clones. However notably, cluster 3 was driven by a TRAV38-2/DV8/TRBV20-1 motif which was identified in the TCR repertoire of predominantly hospitalized moderate and severe/critical COVID-19 patients, though it was also expanded in one mild patient. A subset of COVID-19 patients were also analyzed for their TCRs ability to cross-react to B15/S_919_ and hCoV B15/S_1012_; in alignment with previous findings, cross-reactive TCRs were identified across a number of clusters (#1, 2, 4, 5, 7, 10, 11, 16) including one of the key public motif pairings, TRAV9-2/TRBV7-2 from cluster 1 (*SI Appendix*, Fig. S2*C*).

In alignment with the bias TRBV7-2 gene usage observed, 2 out of 4 primary clusters (#0 and 1) and four additional subclusters (#7, 9, 15, and 17), incorporated the TRBV7-2 gene segment, indicating promiscuous pairing of this TCRβ chain with multiple TCRα chains. This also suggests the TCRβ chain plays a pivotal role in recognition of the B15/S_919_ HLA/peptide complex. Finally, we calculated the probability of generation for TCRα and β chains within each disease group. While none of the disease groups indicated selection of TCRs based on the probability of generation (Fig. 3*C*), cluster 0 had the highest probability of generation for the TCRα chain, suggesting that this public motif may bias the TCR repertoire due to their “easy to generate” nature (Fig. 3*D*).

### No HLA Associations with SARS-CoV-2 Infection across Asian Ancestry Cohorts.

**Fig. 4.**
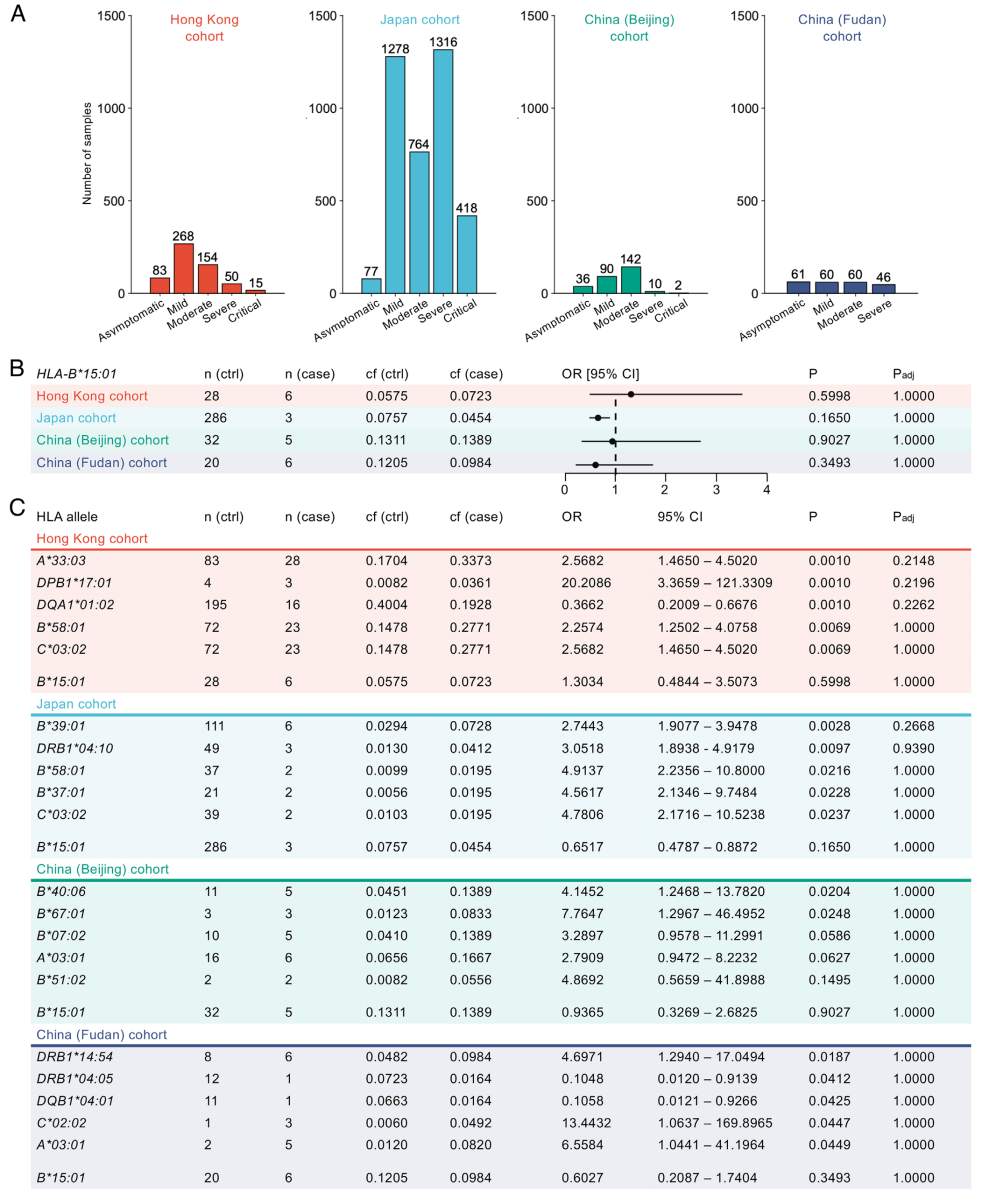
No HLA associations with asymptomatic or symptomatic SARS-CoV-2 infection across four independent Asian ancestry cohorts. (*A*) Asian ancestry cohorts for HLA association analysis. (*B*) Forest-plot depicting odds ratios (OR) and 95% CI with no HLA-B*15:01 association with asymptomatic SARS-CoV-2 infection across four independent Asian ancestry cohorts. (*C*) OR and 95% CI for HLA associations with asymptomatic or symptomatic SARS-CoV-2 infection in four independent Asian ancestry cohorts with Bonferroni correction across alleles. (*A*–*C*) Additional information in *SI Appendix*, Tables S3–S6. ctrl, control; cf, confer; OR, odds ratio; CI, confidence interval; *P*_adj_, adjusted *P* value.

HLA-B*15:01 has previously been associated with asymptomatic SARS-CoV-2 infection in comparison to mild COVID-19 in nonhospitalized individuals of European ancestry (4). Here, we sought to understand whether HLA-B*15:01 also offers protection across different ethnicities, by analyzing four independent cohorts of unvaccinated HLA-typed individuals of Asian ancestry from Hong Kong (24), Japan (25), and China [Beijing (26, 27) and Fudan groups] (Fig. 4*A* and *SI Appendix*, Table S1).

Overall, HLA-B*15:01 was expressed in 12.58% of SARS-CoV-2-infected individuals as heterozygous (Hong Kong: 5.96%, Japan: 13.73%, China (Beijing): 13.21%, and China (Fudan): 11.45%) and 0.61% as homozygous (0%, 0.65%, 1.43%, 0.44%, respectively). Across these cohorts, an average of 22.37% of HLA-B*15:01-expressing individuals developed severe or critical disease, similar to 22.24% in the overall cohorts, demonstrating no clear protective effect (*SI Appendix*, Table S2). In the Hong Kong cohort, HLA-B*15:01 was relatively enriched among individuals reporting asymptomatic infection (frequency = 0.07) compared to those with symptoms (carrier frequency (cf) = 0.06), yielding an odds ratio (OR) of 1.30. However, this association with asymptomatic infection was not statistically significant (95% CI = 0.48 to 3.51, *P* = 0.60, *P*_adj_ = 1) after adjusting for age, gender, and HLA locus (Fig. 4*B* and *SI Appendix*, Table S3). Both cohorts from China (Beijing and Fudan groups) showed no significant bias toward symptomatic or asymptomatic infection among HLA-B*15:01^+^ individuals, resulting in insignificant odds ratios (Fig. 4*B* and *SI Appendix*, Tables S4 and S5). In the Japan cohort (25), we observed an enrichment of HLA-B*15:01^+^ individuals reporting symptomatic infection (symptomatic frequency = 0.07, asymptomatic frequency = 0.05), with an odds ratio of 0.65, albeit this did not reach statistical significance (95% CI = 0.89 to 0.48, *P* = 0.17, *P*_adj_ = 1) (Fig. 4*B* and *SI Appendix*, Table S6). Notably, no HLA alleles were significantly associated with asymptomatic or symptomatic infection in any of the four independent Asian ancestry cohorts after applying Bonferroni correction across all alleles (Fig. 4*C* and *SI Appendix*, Tables S3–S6). These findings suggest that the association of HLA-B*15:01 with asymptomatic infection, as identified by Augusto et al. (4) is not universally applicable and may be defined by ancestry.

## Discussion

Preexisting immunity from high COVID-19 vaccination rates and prior infection has decreased SARS-CoV-2 related severe illness and death. However, emergence of SARS-CoV-2 in early 2020 provided a novel immunological situation where primary infection could be studied at a global level. Prior to our study, the ex vivo B15/S_919_^+^CD8^+^ T cell response following natural SARS-CoV-2 infection across disease severities were ill-defined. Our in-depth quantitative, phenotypic, and clonal profiling of ex vivo epitope-specific T cell responses found consistent frequencies of memory B15/S_919_-specific CD8^+^ T cells prior to and following COVID-19. Conversely, other SARS-CoV-2 CD8^+^ specificities were predominantly naïve in prepandemic samples, and while they gained a prototypical, expanded central memory phenotype following SARS-CoV-2 infection, the frequency of these other memory T cells was not maintained to 12 mo postinfection. We and others have previously observed naïve CD8^+^ T cells in the periphery of immunologically naïve individuals across a number of viral infections including HIV, HCV, and SARS-CoV-2 (13, 14, 16, 18, 28, 29). Our findings align with the cross-reactive nature of the B15/S_919_-specific CD8^+^ T cell response, first identified through shared TCR clonotypes between SARS-CoV-2 and hCoV HKU1/OC43 in SARS-CoV-2-infected and COVID-vaccinated individuals (5). Our data showed activation of central memory B15/S_919_-specific CD8^+^ T cells during acute infection, without an accompanying rise in cell number that typically coincides with primary infection (14, 18, 30). This aligns with findings in influenza infection, where there is no boost in the magnitude of influenza-specific CD8^+^ T cells in peripheral blood following influenza virus infection (15), most likely as virus-specific memory CD8^+^ T cells traffic to the site of infection. Whether memory B15/S_919_-specific CD8^+^ T cells are preferentially recruited into the SARS-CoV-2 response ahead of other naïve SARS-CoV-2 specificities and whether this prior antigen-exposure helps to control COVID-19 resulting in more asymptomatic infections remains a hypothesis. However, our data suggested that while the T cell frequency in the circulation across different specificities were comparable during SARS-CoV-2 infection, prior antigen-exposure provided better maintenance of the cross-reactive B15/S_919_^+^CD8^+^ T cells over time.

Ex vivo tetramer-enrichment in HLA-B*15:01^+^ COVID-19 patients across different disease severity states, from asymptomatic to severe and critical patients, revealed no numerical advantage for the presence of cross-reactive, central memory-like B15/S_919_-specific CD8^+^ T cells. Rather, patients with severe/critical COVID-19 tended to have lower levels of B15/S_919_^+^CD8^+^ T cells in the circulation compared to mildly infected patients. Given that following moderate to critical COVID-19, SARS-CoV-2-specific memory T cells can be enriched at the site of infection compared with the blood (31), this slight decrease in B15/S_919_-specific CD8^+^ T cells with severity might result from preferential recruitment of memory T cells into the site of infection following SARS-CoV-2 infection. However, it is unknown whether severe disease in HLA-B*15:01-expressing patients is associated with dysregulated B15/S_919_-specific T cell responses in the lung or due to lower levels of these cross-reactive CD8^+^ T cells in the circulation prior to SARS-CoV-2 infection.

Our analysis of the B15/S_919_-specific TCRαβ repertoire from COVID-19 patients revealed public cross-reactive clonotypes shared by prepandemic individuals from our cohort and others (4, 5). We identified these public clonotypes and motifs across different COVID-19 severity groups. However, patients with severe/critical illness had reduced clonal expansion of one of the key public TCR pairings (TRAV9-2/TRBV7-2), and instead enrichment of an “alternate” TCR motif (TRAV38-2/DV8/TRBV20-1). Whether these TCR motifs are represented in the TCR repertoire of patients that go on to develop severe/critical COVID-19, or where they go during and following severe/critical infection remains to be determined. Interestingly, prepandemic TCR repertoires specific to B15/S_919_ and B7/N_105_, both associated with milder SARS-CoV-2 infection, shared higher similarity with mild disease compared to severe disease (13). This is particularly interesting given CD8^+^ T cells directed toward these two epitopes appear to have different dynamics associated with SARS-CoV-2 infection. B7/N_105_^+^CD8^+^ T cells are predominantly naïve in prepandemic individuals (14), with the mechanism of protection from severe disease believed to be early recruitment of high-affinity TCR clonotypes from a very diverse TCR repertoire (13). In contrast, B15/S_919_-specific CD8^+^ T cells are antigen-experienced, with a central memory-like phenotype and a more restricted TCR repertoire. Our data suggest less clonal expansion and recruitment of alternate clonotypes into the circulation during and following severe COVID-19. While key public TCR clonotypes may be found at the site of infection, previous work with SARS-CoV-2 and influenza virus infection suggests that the TCR repertoire of the tissue often reflects that of the circulation (32–34). Finally, while TCRs from the severe/critical B15/S_919_^+^CD8^+^ TCR repertoire had a similar probability of generation to those from prepandemic and milder infections, it remains unknown whether these TCRs are of lower affinity or functional capacity, as observed for B7/N_105_^+^CD8^+^ T cell responses in severe COVID-19 (13).

Our study reveals that despite HLA-B*15:01 being associated with asymptomatic infection in European ancestry cohorts, 22.37% of HLA-B*15:01-positive individuals developed critical/severe disease and were hospitalized across four independent cohorts of Asian ancestry, with 4,930 participants. We demonstrate that despite high frequency and central memory-like phenotype in prepandemic samples, B15/S_919_-specific CD8^+^ T cell frequencies and phenotype were generally comparable across disease severity groups. While the frequency and clonal expansions of the key public B15/S_919_-specific TRAV9-2/TRBV7-2 TCR motif decreased with increasing disease severity. Importantly, we show no association between HLA-B*15:01 and asymptomatic infection among our four cohorts of Asian ancestry, suggesting that HLA associations with asymptomatic or severe disease might be relevant to specific populations and HLA combinations. Our work highlights the importance of CD8^+^ T cell responses in disease outcomes and suggests that generating prior cellular immunity as well as boosting robust CD8^+^ T cell responses, such as through new generation vaccines, has the potential to improve patient outcomes.

### Limitations of the Study.

Future studies are needed to define whether the alternate TCR motif identified in our study in severe/critical patients is linked to differences in peptide–HLA affinity or functional capacity of B15/S_919_-specific CD8^+^ T cells in life-threatening disease. The overlap between our prepandemic and post-COVID-19 TCR repertoires suggests the B15/S_919_-specific TCRs observed following COVID-19 were originally induced during a prior hCoV infection. However, as we have no participants in our study who were sampled pre- and post-COVID-19, this cannot be confirmed. In addition, it remains to be determined whether the key public TCRs exist in the TCR repertoire of patients that go on to develop severe/critical COVID-19, and if so, where these T cells go during and following severe/critical infection. While we sequenced between 38 and 271 TCR chains within each disease severity group, our ability to identify prominent clusters and TCR motifs within each disease group may be limited by our sequencing depth. Our sample size, sampled across multiple large cohorts, is pragmatic given this cohort represents a now unique population of primary SARS-CoV-2-infected unvaccinated HLA-B*15:01-expressing patients across disease severity.

## Materials and Methods

### Patient Cohorts and Ethics Statement.

Unvaccinated participants experiencing a primary SARS-CoV-2-infection were recruited in Melbourne (Australia), Hong Kong, and United Kingdom as part of larger respiratory virus infection cohorts. Participants from these cohorts expressing the HLA-B*15:01 allele were included in cellular analyses (*SI Appendix*, Tables S7 and S8). All participants were unvaccinated for the duration of the study, with exception of two 12-mo postinfection samples (*SI Appendix*, Table S8).

Our Australian database of COVID-19 patient and prepandemic or preinfection (SARS-CoV-2 seronegative) cohorts and their HLA class I and class II typing have been described previously (14, 16, 18, 30, 35–40). This study was approved by the Alfred Hospital (no. 280/14), Melbourne Health (HREC/66341/MH-2020), Austin Health (HREC/63201/Austin-2020), Australian Red Cross Lifeblood (2015#08), University of Melbourne (nos. 2056901, 13344, 14013, 25684, 2022-13973-25841-5 and 2020-20782-12450-1), Institutional Review Board HKU/HA Hong Kong West Cluster (UW 20-273, UW 20-169, UW 20-132), Joint Chinese University of Hong Kong-New Territories East Cluster (CREC 2020.229), Institutional Review Board of The Hong Kong University, Hong Kong Island West Cluster of Hospitals (UW 16-254), and the PHOSP-COVID biobank (#20/YH/0225) with approvals from NIHR, Leicester Biomedical Research Centre-Respiratory and Department of Respiratory Sciences, University of Leicester, Glenfield Hospital human research ethics committees. All participants provided written informed consent.

HLA association cohorts, anti-RBD IgG ELISA, ex vivo tetramer enrichment and TCR analysis are described in *SI Appendix*.

## Supplementary Material

Appendix 01 (PDF)

Dataset S01 (XLSX)

Dataset S02 (XLSX)

Dataset S03 (XLSX)

Dataset S04 (XLSX)

Dataset S05 (XLSX)

Dataset S06 (XLSX)

Dataset S07 (XLSX)

## Data Availability

All study data are included in the article and/or supporting information.
