# Supplementary material for: HLA-B*15:01-positive severe COVID-19 patients lack CD8+ T cell pools with highly expanded public clonotypes
Source: Proc Natl Acad Sci U S A. 2025 Sep 2;122(36):e2503145122. doi: 10.1073/pnas.2503145122 (PMC12435269; doi:10.1073/pnas.2503145122)
Supplement: Supplementary file 1 — Appendix 01 (PDF) [file pnas.2503145122.sapp.pdf]

HLA-B*15:01-positive severe COVID-19 patients lack $CD8^+$ T cell pools with highly expanded public clonotypes

METHODS

Participant cohorts and Ethics Statement

Participants from the Melbourne (Australia), Hong Kong and United Kingdom cohorts expressing the HLA-B*15:01 allele were included in the cellular analysis for this study (Tables S7 and S8). COVID-19 patients were 35% female and SARS-CoV-2-infected between March 2020 and December 2022. Patients were assessed for their $CD8^+$ T cell responses over the first ~12 months post primary SARS-CoV-2 infection (range: 0-482 days) (Figure S1A). Pre-pandemic/pre-infection participants were 50% female. PBMCs from Melbourne, Australia were isolated by Ficoll-Paque separation for cellular assays and DNA isolated from granulocytes for HLA typing by the Victorian Transplantation and Immunogenetics Service (Australian Red Cross Lifeblood, Melbourne, Australia), as described (1). PBMCs from Hong Kong, China were isolated by Ficoll Paque density gradient centrifugation using Leucosep tubes[33] for cellular assays. For Hong Kong COVID-19 patients, DNA was isolated for HLA typing by Qiagen DNAeasy kit according to manufacturer's instructions, and typing by the Hospital Authority (Hong Kong SAR). For Hong Kong seronegative adult negative controls, aggregate HLA typing information was provided by the Hong Kong Bone Marrow Donor Registry. For UK COVID-19 patients, PBMCs from the PHOSP-COVID biobank were isolated as previously described (2). Some samples were used following CD14 positive enrichment, with $CD14^-$ cells isolated from the PBMC population using MACS (Miltenyi Biotec, Germany). Briefly, cells were resuspended in MACS buffer and incubated with CD14 microbeads (Miltenyi Biotec) on ice for 15min before passing them through a LS magnetic column (Miltenyi Biotec). The column was washed 3 times with MACS buffer and the recovered cells collected as the CD14 negative fraction. Class I HLA alleles were genotyped using the commercial platform developed by HistoGenetics (Ossining, NY) using sequencing-based typing and NGS methods or Axiom microarrays coupled with imputation (3). For samples with only class I HLA typing (UK and some AUS), HLA-DPB1*04:01 expression was confirmed by tetramer staining. The study was conducted in compliance with the conditions of the ethics committee approval, the NHMRC National Statement on Ethical Conduct in Human Research (2007) and the Note for Guidance on Good Clinical Practice (CPMP/ICH-135/95).

Data from four independent participant cohorts were also included for HLA association studies, all of whom were unvaccinated and tested positive for SARS-CoV-2 for the first time between May 2020 and December 2022. The larger Hong Kong cohort was analysed for disease severity and HLA allele expression (4) (Dataset S3). In addition, two cohorts recruited in China were analysed. For the China (Beijing) cohort (5, 6), written informed consent was obtained from all participants (including guardians of minors). The study was approved by the Ethics Committee of National Institute for Viral Disease Control and Prevention, Chinese Center for Disease Control and Prevention (China CDC) (IVDC2020-021 and IVDC2021-007). The HLA typing was done at 4- or 6-digit resolution by Sanger sequencing or LABType SSO tests; with the 4-digit resolution shown here (Dataset S4). For the China (Fudan) cohort, written informed consent was obtained from all participants and the study was approved by the Shanghai Public Health Clinical Center Ethics Committee (2020-Y008-01). Peripheral blood samples were centrifuged according to the manufacturer's instructions and used to separate mononuclear cells. Genomic DNA was extracted from the PBMC sample of each patient and used for this study as the subjects of HLA genotyping using the next-generation sequencing method, targeting class I (HLA-A, -B, and -C) and class II (HLA-DPA1, -DPB1, -DRB1, -DQA1 and -DQB1) alleles. Each HLA allele was amplified with locus-specific primers using a long-range PCR method. The primers were designed to anneal to conserved regions. DNA libraries of these PCR products were prepared using the transposase-mediated library preparation method with the Nextera DNA Sample Preparation Kit or Nextera XT DNA Library Prep Kit (Illumina). The library was subjected to multiplex sequencing using NovaSeq 6000 platform (PE150 read length) (Illumina). To identify the HLA genotype, sequencing reads were aligned using the CLC Bio Genomics Workbench. The HLA typing was done at 4-digit resolution (Dataset S5). Finally, we also analysed disease severity and HLA expression in an expanded published cohort recruited in Japan (Dataset S6) (7). Here, we report on individual's disease severity status based on NIH scores

(8). Throughout our study, participants were designated as "asymptomatic" only if they had a positive SARS-CoV-2 PCR result and remained symptom-free for their full disease course.

Detection of ancestral SARS-CoV-2 RBD-specific IgG antibodies

Plasma anti-RBD IgG antibody titres were determined for participants included in the cellular analysis for this study. Plasma ancestral anti-SARS-CoV-2 RBD protein IgG levels for Melbourne participants were determined by ELISA as previously described in detail (1). Similarly, plasma antibodies from participants from Hong Kong, China were assessed by IgG ELISA as previously described (9), with the modification of using RBD (Sino Biological) ancestral SARS-CoV-2 protein.

Spike and non-spike epitope-specific tetramer$^+$ T cell responses

HLA class I and class II tetramers were generated by the Rossjohn Laboratory, Monash University, Australia, as previously described, including HLA-A*02:01/S_{269} (YLQPRTFLL), HLA-A*03:01/N_{361} (KTFPPTEPK), HLA-A*24:02/S_{1208} (QYIKWPWYI), HLA-B*07:02/N_{105} (SPRWYFYYL), HLA-B*15:01/S_{919} (NQKLIANQF), HLA-B*40:01/N_{322} (MEVTPSGTWL) and HLA-DPA1*01:03/DPB1*04:01/S_{167} (TFEYVSQPFLMDLE) (1, 10-13). All epitopes examined originate from the ancestral SARS-CoV-2 spike or nucleocapsid and are highly conserved across SARS-CoV-2 variants of concern (VOC) strains(11). The HLA-B15-restricted hCoV tetramer (HLA-B*15:01/S_{1012}; NQKLIANAF) was also generated by the Rossjohn Laboratory.

Cryopreserved PBMCs (0.6-18x10^6) were stained with class I HLA-B*15:01/S_{919} tetramer on PE, and if feasible, an additional class I SARS-CoV-2 tetramer on APC and DPB4/S_{167} class II tetramer on PE before undergoing tetramer-associated magnetic enrichment (TAME) as described (1, 11, 14, 15). Tetramers on PE were exclusively stained on CD8$^+$ T cells for HLA-B*15:01/S_{919} and on CD4$^+$ T cells for DPB1*04:01/S_{167}. Enriched tetramer$^+$ T cells were indexed single-cell sorted on a BD FACSAria III using the software BD FACS DIVA v8.0.1 for TCR analysis essentially as described (1, 11). Flow cytometry data were analyzed using FlowJo v10 software (Figure S3). Tetramer-positive frequencies corresponding to <10 tetramer$^+$ events are displayed as open symbols and are not including in the phenotypic and activation analysis. For samples run in duplicate, averaged tetramer$^+$ T cell frequencies were plotted.

TCRαβ analysis

CDR3α and CDR3β loops from single cells were amplified using multiplex-nested RT-PCR (1, 16) and analyzed by IMGT/V-QUEST. Circos plots were made using circlize v.0.4.16 (17) in R v.4.4.1 (18). Alluvial plots were made using the ggalluvial v.0.12.5 package (19). Paired alpha and beta TCR sequences were analysed by tcrdist3 (20, 21) in Python v.3.8.16 for determination of pairwise distances between clonotypes and probabilities of generation (P_{gen}). Clonotype networks with edges representing ≤ 120 tcrdist units were generated with NetworkX v.3.1 and visualised with Cytoscape v.3.10.2 (22). Raw and background subtracted sequence logo plots were generated using palmotif v.0.4, with TCR beta backgrounds sampled from cord blood sequences (23) and alpha backgrounds from a repertoire simulated using OLGA (24, 25).

Statistical analysis

Statistical significance of nonparametric datasets (two-tailed) were determined using GraphPad Prism v9 software. Wilcoxon signed-rank test (paired 2 groups), Kruskal-Wallis test (unmatched) with Dunn's multiple comparisons, Tukey's multiple comparison test, Simpson's Diversity Index, Fisher's exact test and odds ratios (OD) with 95% confidence intervals (CI) were used and indicated in the figure legends. *$P \leq 0.05$, **$P \leq 0.01$, ***$P \leq 0.001$, **** $P \leq 0.0001$.

HLA association analysis

We investigated the association between asymptomatic or symptomatic SARS-CoV-2 infection with eight HLA loci (HLA-A, -B, -C, -DRB1, -DQA1, -DQB1, -DPA1, and -DPB1) for the Hong Kong, Japan and China (Fudan) cohorts and two loci (HLA-A and -B) in the China (Beijing) cohort. Using a dominant inheritance model, we defined carrier frequency (cf) as the proportion of individuals in the cohort carrying at least one copy of the allele of interest. Asymptomatic infections were treated as cases, while symptomatic infections served as controls. For the cohorts from China (Beijing and Fudan) and Hong Kong, we tested associations while controlling for confounding factors such as age and gender by employing a generalized linear regression

model using the fitglm function in MATLAB®. In the Japan cohort, where full genomic data was available, we utilized SAIGE (26) for association testing, incorporating age, sex, and the top two principal components of the genomic data (including MHC regions) as covariates in the regression model. To account for multiple comparisons across loci, we applied the Bonferroni method to adjust the P values for each association tested in the cohorts.

PHOSP acknowledgements

This PHOSP study would not be possible without all the participants who have given their time and support. We thank all the participants and their families. We thank the many research administrators, health-care and social-care professionals who contributed to setting up and delivering the study at all of the 65 NHS trusts/Health boards and 25 research institutions across the UK, as well as all the supporting staff at the NIHR Clinical Research Network, Health Research Authority, Research Ethics Committee, Department of Health and Social Care, Public Health Scotland, and Public Health England,

and support from the ISARIC Coronavirus Clinical Characterisation Consortium. We thank Kate Holmes at the NIHR Office for Clinical Research Infrastructure (NOCRI) for her support in coordinating the charities group. The PHOSP-COVID industry framework was formed to provide advice and support in commercial discussions, and we thank the Association of the British Pharmaceutical Industry as well NOCRI for coordinating this. We are very grateful to all the charities that have provided insight to the study: Action Pulmonary Fibrosis, Alzheimer's Research UK, Asthma + Lung UK, British Heart Foundation, Diabetes UK, Cystic Fibrosis Trust, Kidney Research UK, MQ Mental Health, Muscular Dystrophy UK, Stroke Association Blood Cancer UK, McPin Foundations, and Versus Arthritis. We thank the NIHR Leicester Biomedical Research Centre patient and public involvement group and Long Covid Support.

Supplemental Tables

Table S1: Four independent Asian ancestry cohorts for HLA association analysis

Features \ Cohort		Hong Kong(4)	Japan(7)	China (Beijing)(5, 6)	China (Fudan)
Loci data		A, B, C, DRB1, DQA1, DQB1, DPA1, DPB1	A, B, C, DRB1, DQA1, DQB1, DPA1, DPB1	A, B	A, B, C, DRB1, DQA1, DQB1, DPA1, DPB1
Infection outcome	Asymptomatic	83	77	36	61
	Mild	268	1,316	90	60
	Moderate	154	418	142	60
	Severe	50	1,278	10	46
	Critical	15	764	2	0
	Total	570	3,853	280	227

Table S2: Disease severity across four independent Asian ancestry cohorts

Cohort	Overall cohort				HLA-B*15:01-positive individuals			
	Asymptomatic /Mild/Moderate	Severe/Critical	Total	% Severe/Critical	Asymptomatic /Mild/Moderate	Severe/Critical	Total	% Severe/Critical
Hong Kong	505	65	570	11.40	30	4	34	11.76
Japan	1811	2,042	3,853	53.00	242	312	554	56.32
China (Beijing)	268	12	280	4.29	35	2	37	5.41
China (Fudan)	181	46	227	20.26	21	4	25	16
Average				22.24				22.37

Table S3. HLA associations with asymptomatic or symptomatic SARS-CoV-2 infection in Hong Kong cohort with Bonferroni correction for all alleles

HLA allele	OR	OR CI (left)	OR CI (right)	P-value	P-value (adj)	cf (ctrl)	cf (case)	n (ctrl)	n (case)
A*33:03	2.5682	1.4650	4.5020	**0.0010**	0.2148	0.1704	0.3373	83	28
DPB1*17:01	20.2086	3.3659	121.3309	**0.0010**	0.2196	0.0082	0.0361	4	3
DQA1*01:02	0.3662	0.2009	0.6676	**0.0010**	0.2262	0.4004	0.1928	195	16
B*58:01	2.2574	1.2502	4.0758	**0.0069**	1.0000	0.1478	0.2771	72	23
C*03:02	2.5682	1.4650	4.5020	**0.0069**	1.0000	0.1478	0.2771	72	23
B*15:01	1.3034	0.4844	3.5073	0.5998	1.0000	0.0575	0.0723	28	6

Results are shown for the top five most significant HLA associations and for HLA-B*15:01.
The total number of symptomatic (ctrl) and asymptomatic (case) individuals in the cohort was n=487 and n=83, respectively.

P-value (adj): Bonferroni-corrected P-value applied across all alleles (n=217); cf: carrier frequency, representing the frequency of individuals in the cohort who carry at least one copy of the allele of interest.

Table S4. HLA associations with asymptomatic or symptomatic SARS-CoV-2 infection in China (Beijing) cohort with Bonferroni correction for HLA-A and B alleles

HLA allele	OR	OR CI (left)	OR CI (right)	P-value	P-value (adj)	cf (ctrl)	cf (case)	n (ctrl)	n (case)
B*40:06	4.1452	1.2468	13.7820	**0.0204**	1.0000	0.0451	0.1389	11	5
B*67:01	7.7647	1.2967	46.4952	**0.0248**	1.0000	0.0123	0.0833	3	3
B*07:02	3.2897	0.9578	11.2991	0.0586	1.0000	0.0410	0.1389	10	5
A*03:01	2.7909	0.9472	8.2232	0.0627	1.0000	0.0656	0.1667	16	6
B*51:02	4.8692	0.5659	41.8988	0.1495	1.0000	0.0082	0.0556	2	2
B*15:01	0.9365	0.3269	2.6825	0.9027	1.0000	0.1311	0.1389	32	5

Results are shown for the top five most significant HLA associations and for HLA-B*15:01.
The total number of symptomatic (ctrl) and asymptomatic (case) individuals in the cohort was n=244 and n=36, respectively.
P-value (adj): Bonferroni-corrected P-value applied across all alleles with data (HLA-A and -B; n=85); cf: carrier frequency.

Table S5. HLA associations with asymptomatic or symptomatic SARS-CoV-2 infection in China (Fudan) cohort with Bonferroni correction for all alleles

HLA allele	OR	OR CI (left)	OR CI (right)	P-value	P-value (adj)	cf (ctrl)	cf (case)	n (ctrl)	n (case)
DRB1*14:54	4.6971	1.2940	17.0494	**0.0187**	1.0000	0.0482	0.0984	8	6
DRB1*04:05	0.1048	0.0120	0.9139	**0.0412**	1.0000	0.0723	0.0164	12	1
DQB1*04:01	0.1058	0.0121	0.9266	**0.0425**	1.0000	0.0663	0.0164	11	1
C*02:02	13.4432	1.0637	169.8965	**0.0447**	1.0000	0.0060	0.0492	1	3
A*03:01	6.5584	1.0441	41.1964	**0.0449**	1.0000	0.0120	0.0820	2	5
B*15:01	0.6027	0.2087	1.7404	0.3493	1.0000	0.1205	0.0984	20	6

Results are shown for the top five most significant HLA associations and for HLA-B*15:01.
The total number of symptomatic (ctrl) and asymptomatic (case) individuals in the cohort was n=166 and n=61, respectively.
P-value (adj): Bonferroni-corrected P-value applied across all alleles (n=192); cf: carrier frequency.

Table S6. HLA associations with asymptomatic or symptomatic SARS-CoV-2 infection in Japan cohort with Bonferroni correction for all alleles

HLA allele	OR	OR CI (left)	OR CI (right)	P-value	P-value (adj)	cf (ctrl)	cf (case)	n (ctrl)	n (case)
B*39:01	2.7443	3.9478	1.9077	**0.0028**	0.2668	0.0294	0.0728	111	6
DRB1*04:10	3.0518	4.9179	1.8938	**0.0097**	0.9390	0.0130	0.0412	49	3
B*58:01	4.9137	10.8000	2.2356	**0.0216**	1.0000	0.0099	0.0195	37	2
B*37:01	4.5617	9.7484	2.1346	**0.0228**	1.0000	0.0056	0.0195	21	2
C*03:02	4.7806	10.5238	2.1716	**0.0237**	1.0000	0.0103	0.0195	39	2
B*15:01	0.6517	0.8872	0.4787	0.1650	1.0000	0.0757	0.0454	286	3

Results are shown for the top five most significant HLA associations and for HLA-B*15:01.
The total number of symptomatic (ctrl) and asymptomatic (case) individuals in the cohort was n=3,776 and n=77, respectively.
P-value (adj): Bonferroni-corrected P-value applied across all alleles (n=97); cf: carrier frequency.
SAIGE was used for association testing, with age, sex, and top two PCs of patients' genome data (including MHC regions) incorporated into the regression model as covariates(26). The latter factor considers sample relatedness while association testing.

Table S7. Severity group demographics for cellular assays

	Pre-Pandemic	Asymptomatic	Mild	Moderate	Severe/Critical
Number of individuals, *n*	14	3	14	15	13
Age, mean (range)	42 (24-68)	60 (31-91)	52 (25-68)	51 (26-70)	58 (34-75)
Female, *n* (%)	6 (50)	1 (33%)	9 (64%)	1 (6%)	5 (38%)
Days post diagnosis, mean (range)	NA	96 (1-185)	166 (1-482)	132 (1-431)	110 (5-405)
Source, *n* (%)					
Australia	12 (86%)	1 (33%)	5 (50%)	2 (13%)	3 (23%)
Hong Kong	2 (14%)	2 (66%)	5 (50%)	8 (53%)	4 (31%)
United Kingdom	0 (0%)	0 (0%)	0 (0%)	5 (33%)	6 (46%)

Table S8. Cohort demographics for cellular assays

Paper Code	Cohort	NIH Severity Score	Gender	Age	Diagnosis date[a] (mm/yyyy)	Days post diagnosis	HLA-A	HLA-B	HLA-DPB1

1	AUS	-	F	29	pre-pandemic	pre-pandemic	**02:01**	**15:01**, 44:02	**04:01**[d]
2	AUS	-	-	48	pre-pandemic	pre-pandemic	01:01, 11:01	14:02, **15:01**	**04:01**[d]
3	AUS	-	-	45	pre-pandemic	pre-pandemic	03:01, 11:01	**07:02, 15:01**	**04:01**[d]
4	AUS	-	M	60	pre-pandemic	pre-pandemic	**02:01**, 24:02	**15:01**, 35:02	**04:01**[d]
5	AUS	-	F	50	pre-pandemic	pre-pandemic	**02:01**, 03:01	**15:01**, 47:01	**04:01**, 04:02
6	AUS	-	M	35	pre-pandemic	pre-pandemic	02:53N, 03:01	**07:02**, **15:01**	
7	AUS	-	M	68	pre-pandemic	pre-pandemic	01:01; **03:01**	08:01, **15:01**	
8	AUS	-	F	59	pre-pandemic	pre-pandemic	**03:01**, 24:02	**15:01**, 38:01	**04:01**[d]
9	AUS	-	M	24	pre-pandemic	pre-pandemic	02:05, **03:01**	**15:01**, 50:01	**04:01**, 104:01
10	HK	-	F	33	pre-pandemic	pre-pandemic	11:01, **24:02**	**15:01**, 40:01	04:05, 15:01
11	HK	-	M	26	pre-pandemic	pre-pandemic	11:01	**15:01**, **40:01**	04:03, 16:02
12	AUS	-	F	36	pre-pandemic	pre-pandemic	01:01, **03:01**	08:01, **15:01**	
13	AUS	-	F	24	pre-pandemic	pre-pandemic	**02:01**, 03:01	**15:01**, 44:02	03:01, 06:01
14	HK	-	M	47	pre-pandemic	pre-pandemic	03:01, 26:01	**07:02**, **15:01**	
15	HK	1	M	58	01/2021	1,50	11:01, 30:01	13:02, **15:01**	05:01, 17:01
16	AUS	1	F	91	08/2020	66, 185	26:01, 26:01	**15:01**, 38:01	02:01, **04:01**
17	HK	1	M	31	03/2020	180	02:03, 11:01	**15:01**, 15:02	02:01, 03:01
18	HK	2	F	25	08/2020	1, 59, 167	02:07, 11:01	**15:01**, 46:01	05:01
19	HK	2	F	68	02/2021	1, 42, 178, 367[c]	11:01, 33:03	**15:01**, 58:01	09:01, 104:01
20	HK	2	M	63	07/2020	6, 35, 192, 357	11:01, 11:01	**15:01, 40:01**	05:01
21	HK	2	F	62	02/2021	8, 47, 174, 379[c]	11:01, **24:02**	**15:01**, 40:06	14:01, 41:01
22	HK	2	F	42	12/2020	12, 54, 175	02:06, 11:01	**15:01, 40:01**	02:01, 05:01
23	AUS	2	F	65	03/2020	38, 236, 383	**02:01**, 33:01	14:02, **15:01**	02:01, 10:01
24	AUS	2	M	49	03/2020	49, 210, 458	**02:01**, 03:01	**15:01**, 44:02	04:02
25	HK	2	M	31	03/2020	51, 177	**02:01**	**15:01, 40:01**	02:01, 05:01
26	AUS	2	F	54	08/2020	52, 172	**02:01**, 03:01	**15:01**, 40:01	02:01, 04:01
27	AUS	2	F	58	08/2020	53, 191	**02:01**	07:02, **15:01**	04:02
28	AUS	2	M	56	03/2020	61, 229, 482	**02:01**	08:01, **15:01**	01:01, 04:01
29	HK	2	F	37	12/2020	67	**02:01**, 03:01	**15:01**, 35:01	02:01, 04:01
30	AUS	2	F	61	03/2020	70, 201, 406	02:05, 03:01	**15:01**, 50:01	04:01
31	AUS	2	M	60	03/2020	204, 414	03:01, 30:01	**15:01**, 44:03	02:01, 16:01
32	AUS	3	M	38	04/2020	1, 36	**02:01**	**15:01**, 37:01	02:01, **04:01**

33	HK	3	M	68	08/2020	2, 60, 168, 355	11:01	**15:01**, 15:02	05:01
34	HK	3	M	50	09/2020	3, 44, 383	02:07, 11:01	**15:01**, 67:01	02:01, 05:01
35	HK	3	M	70	11/2020	10, 52, 431	**02:01**, 02:03	**15:01**, 38:02	02:01, 48:01
36	HK	3	M	26	07/2020	11, 56, 171	11:01, **24:02**	**15:01**, 40:01	05:01
37	AUS	3	F	36	07/2020	12, 17	**02:01**, 68:01	**15:01**, 39:06	03:01, 138:01
38	HK	3	M	68	07/2020	31	02:07, 11:01	**15:01**, 46:01	05:01
39	HK	3	M	29	07/2020	43, 172, 361	**02:01**, 02:07	**15:01**, 46:01	02:02, **04:01**
40	HK	3	M	64	07/2020	65, 198	11:01, **24:02**	**15:01**, 48:01	05:01, 135:01
41	HK	3	M	51	12/2020	65, 185	**24:02**, 33:03	**15:01**, 58:01	02:02, 13:01
42	UK	3	M	55	§	76	**02:01**, 29:01	**15:01**, 50:01	**04:01**[d]
43	UK	3	M	51	§	145	01:01, 29:02	08:01, 15:01	
44	UK	3	M	65	§	175	**02:01**, 25:01	**15:01**, 18:01	**04:01**[d]
45	UK	3	M	46	§	304	**24:02**	**15:01**, 57:01	**04:01**[d]
46	UK	3	M	54	§	338	01:01, **02:01**	08:01, **15:01**	**04:01**[d]
47	HK	5	M	74	08/2020	2	11:01	**15:01**, 15:02	05:01
48	AUS	4	M	75	08/2020	5, 27	**02:01**	**15:01**, 57:01	04:01
49	AUS	4	F	50	08/2020	11	**02:01**, 68:01	**15:01**, 40:01	04:01
50	HK	4	F	40	08/2020	12	11:01, 33:03	**15:01**, 58:01	05:01
51	AUS	4	M	65	07/2020	12, 405	03:01, 11:01	**15:01**, 44:03	04:01, 11:01
52	HK	4	F	64	12/2020	53, 175	**02:01**, 02:07	**15:01**, 46:01	05:01, 14:01
53	HK	5	F	34	05/2020	40, 65	11:01, **24:02**	**15:01**, 35:01	02:01, 05:01
54	UK	4+	M	74	[b]	80	01:01, **24:02**	**15:01**, 53:01	
55	UK	4+	M	68	[b]	119	**02:01**, 26:01	**15:01**, 44:02	**04:01**[d]
56	UK	4+	M	42	[b]	195	01:01, **02:01**	**15:01**, 39:06	**04:01**[d]
57	UK	4+	M	52	[b]	196	01:01, **02:01**	08:01, **15:01**	**04:01**[d]
58	UK	4+	M	60	[b]	234	01:01, **24:02**	**15:01**, 57:01	
59	UK	4+	F	55	[b]	239	**02:01**, 03:01	**15:01**, 35:01	**04:01**[d]

[a] Diagnosis by symptom onset or SARS-CoV-2 PCR$^+$
[b] UK patients were diagnosed between 03/2020 and 01/2021, severity was not designated between NIH 4 or 5
[c] Sample 1 month post SARS-CoV-2 vaccination
[d] HLA confirmed by tetramer staining
Bold = SARS-CoV-2 epitope examined
AUS = Australia; HK = Hong Kong; UK = United Kingdom

Datasets

Dataset S1: TCR sequences per donor specific to B15/S_{919}, A2/S_{269} and DPB4/S_{167} T cells
Dataset S1 found in excel document: Rowntree B15 COVID_DatasetS1

Dataset S2: PHOSP-COVID Collaborative Group Authors
Dataset S2 found in excel document: Rowntree B15 COVID_DatasetS2

Dataset S3: Hong Kong cohort for HLA analysis

Dataset S3 found in excel document: Rowntree B15 COVID_DatasetS3

Dataset S4: China (Beijing) cohort for HLA analysis
Dataset S4 found in excel document: Rowntree B15 COVID_DatasetS4

Dataset S5: China (Fudan) cohort for HLA analysis
Dataset S5 found in excel document: Rowntree B15 COVID_DatasetS5

Dataset S6: Full HLA association analysis for Japan cohort
Dataset S6 found in excel document: Rowntree B15 COVID_DatasetS6

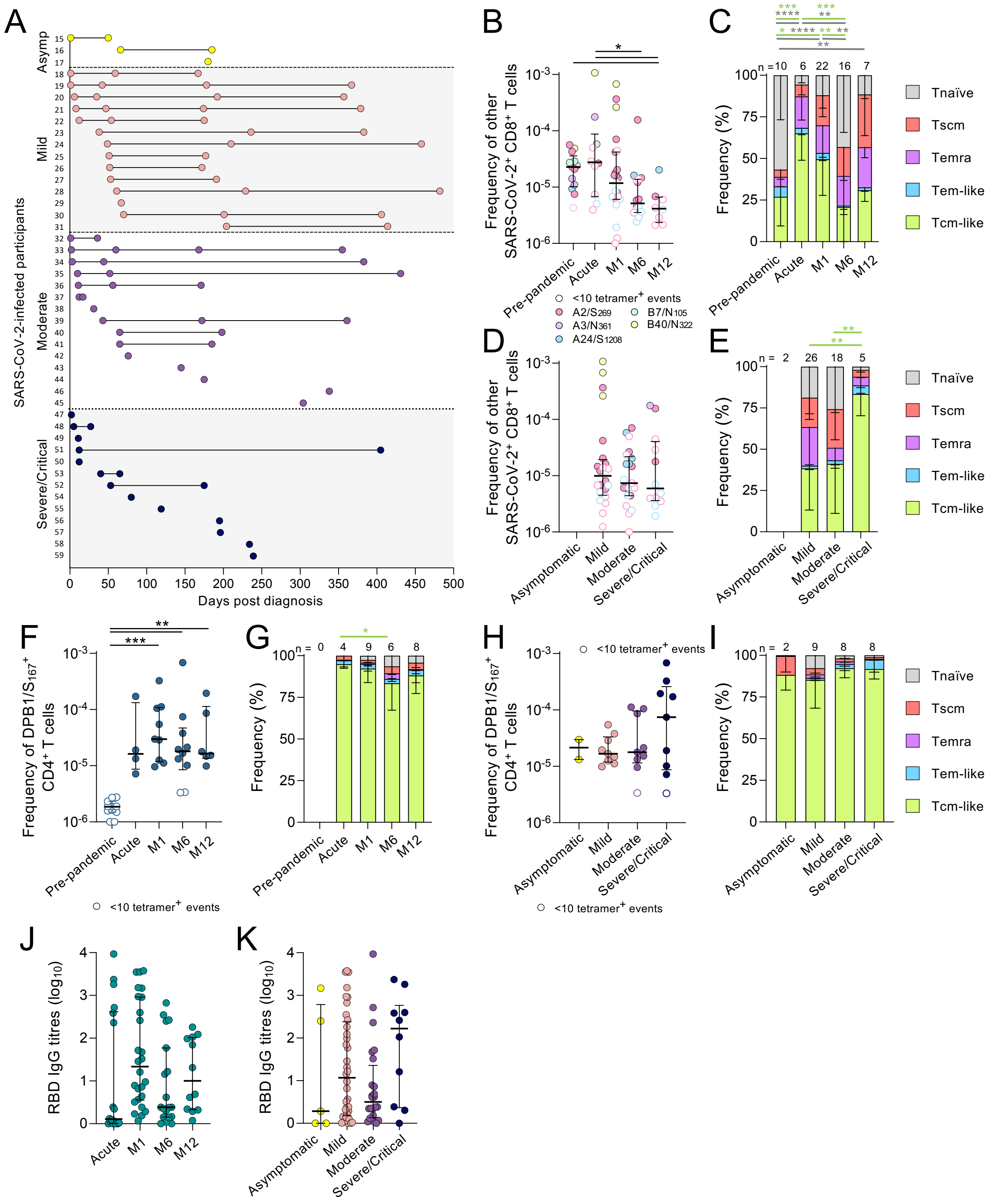

Rowntree *et al.* Figure S1

Figure S1: Other SARS-CoV-2⁺ CD8⁺ and CD4⁺ T cells. (A) Sampling timepoints. (B) Frequency of other SARS-CoV-2$^+$CD8$^+$ T cells pre-pandemic and across time post infection, median with IQR. (C) Memory phenotype profiles across time for other SARS-CoV-2$^+$CD8$^+$ T cells, mean with SD. (D) Frequency of other SARS-CoV-2$^+$CD8$^+$ T cells across disease severity groups, median with IQR. (E) Phenotype profiles across disease severity groups for other SARS-CoV-2$^+$CD8$^+$ T cells, mean with SD. (F) Frequency of DPB4/S$_{167}$$^+$CD4$^+$ T cells pre-pandemic and across time post infection, median with IQR. (G) Phenotype profiles of DPB4/S$_{167}$$^+$CD4$^+$ T cells across time, mean with SD. (H) Frequency of DPB4/S$_{167}$$^+$CD4$^+$ T cells across disease severity groups, median with IQR. (I) Phenotype profiles across disease severity groups for DPB4/S$_{167}$$^+$CD4$^+$ T cells, mean with SD. Endpoint IgG titers of ancestral RBD antibodies across (J) time post infection and (K) disease severity groups. Statistical significance determined by (B, D, F, H, J, K) Dunn's multiple comparisons test and (C, E, G, I) Tukey's multiple comparisons test. *$P \leq 0.05$, **$P \leq 0.01$, ***$P \leq 0.001$, **** $P \leq 0.0001$. The frequency of tetramer$^+$ T cells are shifted by 10^{-6} (i.e. no detected tetramer$^+$ events displayed as 10^{-6}) to allow for visibility on the logarithmic y axis. Any samples with <10 tetramer$^+$ events are shown as open symbols and only samples with 10 or more tetramer$^+$ events are included in the phenotypic analysis (C, E, G, H).

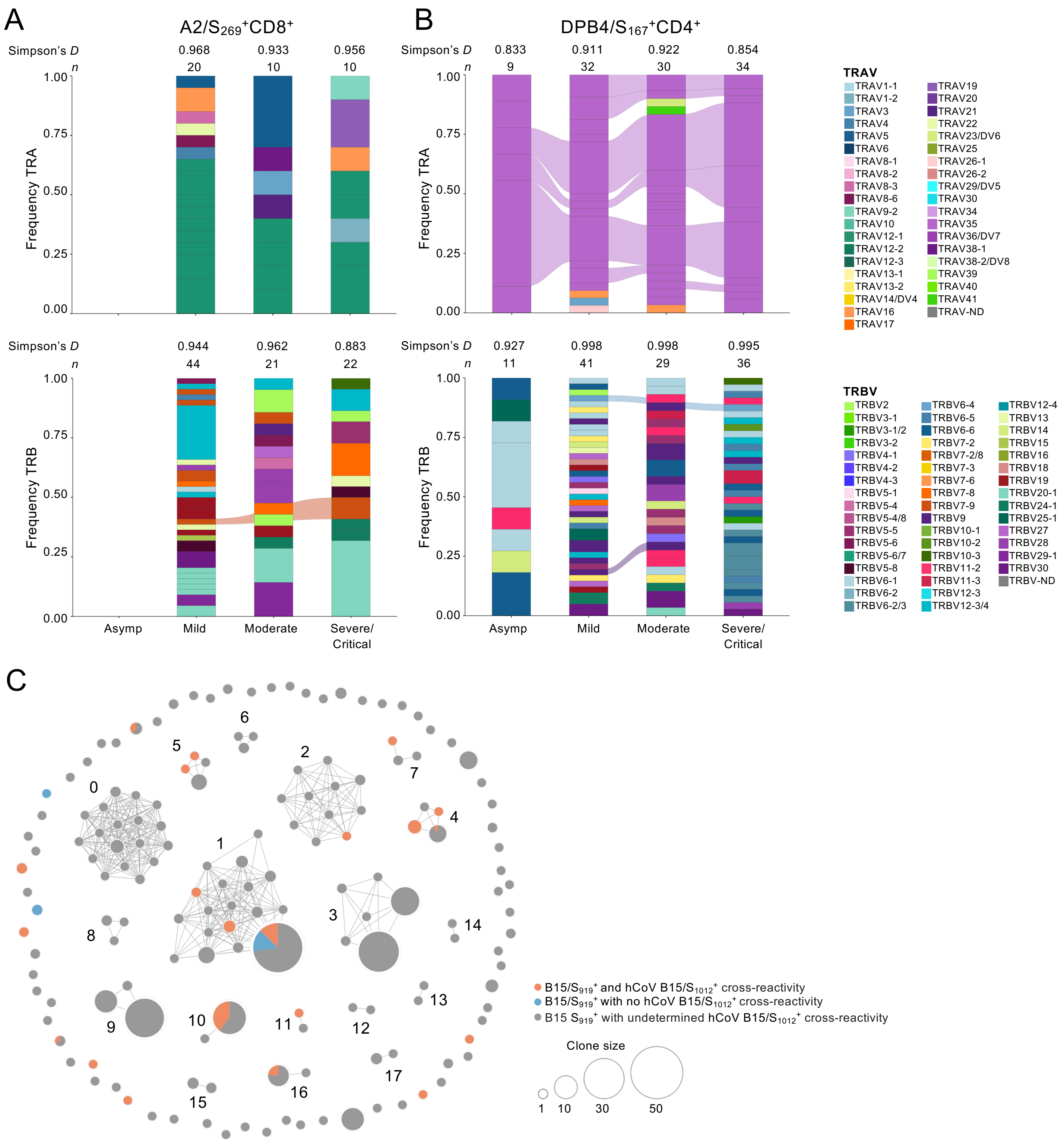

Rowntree *et al*. Figure S2

Figure S2: TCR repertoires associated with A2/S_{269}, DPB4/S_{167} and hCoV B15/S_{1012}. Alluvial plots showing frequency of TRA and TRB gene usage in (A) A2/S_{269}-specific and (B) DPB4/S_{167}-specific TCR repertoires (n=sequences). Connections between bars represent shared CDR3 usage between individuals of different COVID-19 severity. Colours represent variable gene segment usage, while divisions represent TCRαβ clonotypes with the same CDR3 sequence. Statistical significance determined by Simpson's Diversity Index. (C) Network analysis of paired B15/S_{919}-specific TCRαβ clonotypes coloured by cross-reactivity with hCoV B15/S_{1012} (orange).

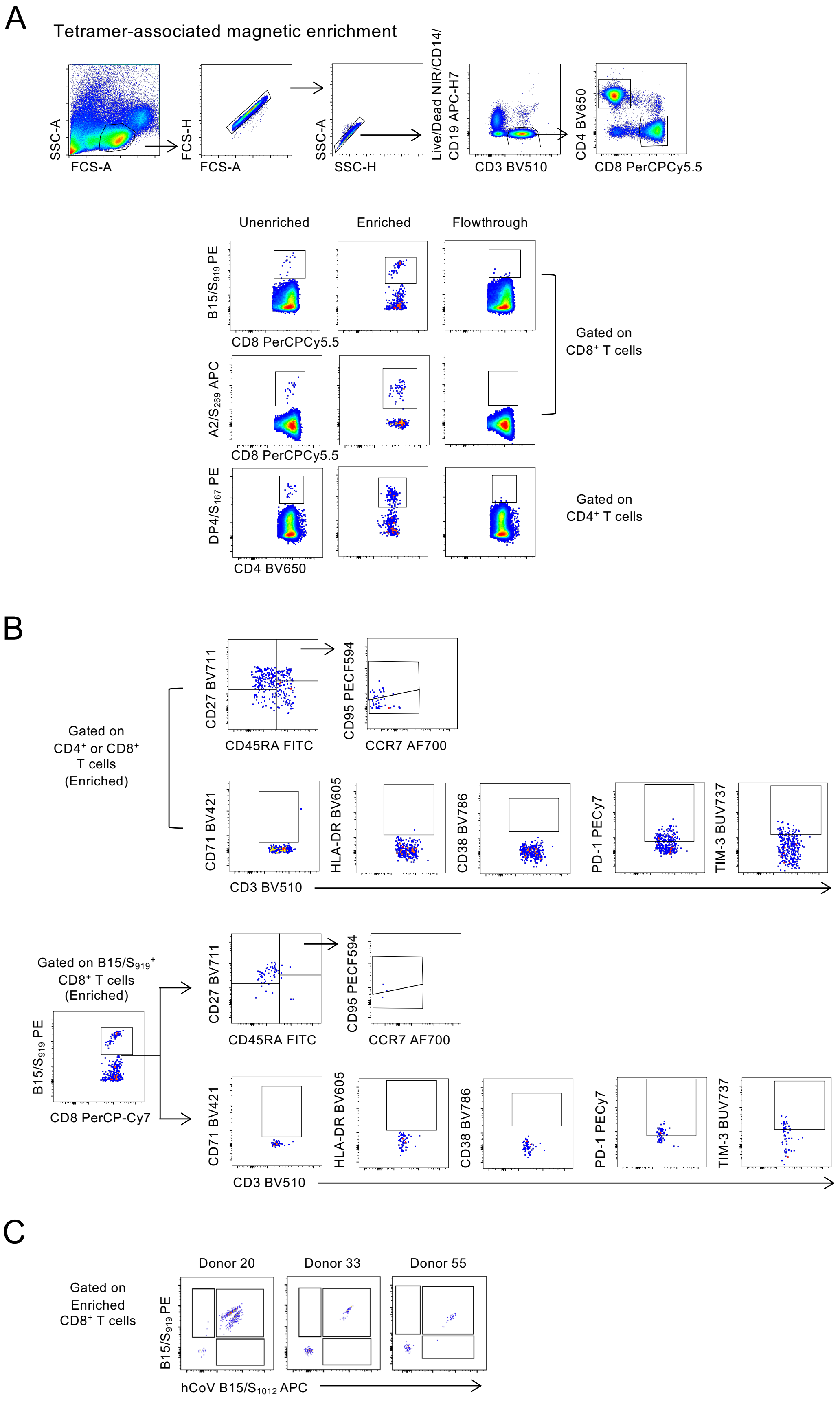

Rowntree *et al*. Figure S3

Figure S3: Gating strategy of TAME assay. (A) Gating workflow for measuring enriched tetramer-specific $CD8^+$ and $CD4^+$ T cells. (B) Gating of memory and activation phenotypes on enriched $CD8^+$ or $CD4^+$ T cells (top panels) and tetramer$^+$ T cells (bottom panels). (C) Representative plots of $B15/S_{919}$ PE and hCoV $B15/S_{1012}$ APC co-staining.

References

1. T. H. O. Nguyen *et al.*, CD8+ T cells specific for an immunodominant SARS-CoV-2 nucleocapsid epitope display high naive precursor frequency and TCR promiscuity. *Immunity* **54**, 1066-1082.e1065 (2021).
2. J. M. Lord *et al.*, Accelerated immune ageing is associated with COVID-19 disease severity. *Immunity & Ageing* **21**, 6 (2024).
3. A. Dilthey *et al.*, Multi-population classical HLA type imputation. *PLoS Comput Biol* **9**, e1002877 (2013).
4. H. Gu *et al.*, Genomic epidemiology of SARS-CoV-2 under an elimination strategy in Hong Kong. *Nat Commun* **13**, 736 (2022).
5. X. Wang *et al.*, Rare peptide anchors of HLA class I alleles contribute to the COVID-19 disease severity and T cell memory. *Biosafety and Health* **5**, 355-362 (2023).
6. J. Zhang *et al.*, One-Year Sustained Cellular and Humoral Immunities in Coronavirus Disease 2019 (COVID-19) Convalescents. *Clin Infect Dis* **75**, e1072-e1081 (2022).
7. H. Namkoong *et al.*, DOCK2 is involved in the host genetics and biology of severe COVID-19. *Nature* **609**, 754-760 (2022).
8. COVID-19 Treatment Guidelines Panel (2023) Coronavirus Disease 2019 (COVID-19) Treatment Guidelines. (National Institutes of Health (NIH)).
9. C. A. Cohen *et al.*, Antibody Fc receptor binding and T cell responses to homologous and heterologous immunization with inactivated or mRNA vaccines against SARS-CoV-2. *Nature Communications* **15**, 7358 (2024).
10. J. R. Habel *et al.*, Suboptimal SARS-CoV-2−specific CD8+ T cell response associated with the prominent HLA-A*02:01 phenotype. *Proc Natl Acad Sci USA* **117**, 24384-24391 (2020).
11. L. C. Rowntree *et al.*, SARS-CoV-2-specific T cell memory with common TCRαβ motifs is established in unvaccinated children who seroconvert after infection. *Immunity* **55**, 1299-1315.e1294 (2022).
12. T. H. O. Nguyen *et al.*, Robust SARS-CoV-2 T cell responses with common TCRαβ motifs toward COVID-19 vaccines in patients with hematological malignancy impacting B cells. *Cell Rep Med* **4**, 101017 (2023).
13. P. A. Mudd *et al.*, SARS-CoV-2 mRNA vaccination elicits a robust and persistent T follicular helper cell response in humans. *Cell* **185**, 603-613.e615 (2022).
14. W. Zhang *et al.*, Robust and prototypical immune responses toward COVID-19 vaccine in First Nations peoples are impacted by comorbidities. *Nat Immunol* **24**, 966-978 (2023).
15. C. E. van de Sandt *et al.*, Newborn and child-like molecular signatures in older adults stem from TCR shifts across human lifespan. *Nature Immunology* 10.1038/s41590-023-01633-8 (2023).
16. S. A. Valkenburg *et al.*, Molecular basis for universal HLA-A*02:01-restricted CD8+ T-cell immunity against influenza viruses. *Proceedings of the National Academy of Sciences USA* 10.1073/pnas.1603106113, 201603106 (2016).
17. Z. Gu, L. Gu, R. Eils, M. Schlesner, B. Brors, Circlize implements and enhances circular visualization in R. *Bioinformatics* **30**, 2811-2812 (2014).
18. R Core Team (2022) R: A language and environment for statistical computing. in *R Foundation for Statistical Computing* (Vienna, Austria).
19. J. C. Brunson, Q. D. Read (2023) ggalluvial: Alluvial Plots in 'ggplot2'. in *R package version 0.12.5*.
20. P. Dash *et al.*, Quantifiable predictive features define epitope-specific T cell receptor repertoires. *Nature* **547**, 89-93 (2017).
21. K. Mayer-Blackwell *et al.*, TCR meta-clonotypes for biomarker discovery with tcrdist3 enabled identification of public, HLA-restricted clusters of SARS-CoV-2 TCRs. *Elife* **10** (2021).
22. P. Shannon *et al.*, Cytoscape: a software environment for integrated models of biomolecular interaction networks. *Genome Res* **13**, 2498-2504 (2003).
23. O. V. Britanova *et al.*, Dynamics of Individual T Cell Repertoires: From Cord Blood to Centenarians. *J Immunol* **196**, 5005-5013 (2016).
24. Z. Sethna, Y. Elhanati, C. G. Callan, A. M. Walczak, T. Mora, OLGA: fast computation of generation probabilities of B- and T-cell receptor amino acid sequences and motifs. *Bioinformatics* **35**, 2974-2981 (2019).

25. Q. Marcou, T. Mora, A. M. Walczak, High-throughput immune repertoire analysis with IGoR. *Nat Commun* **9**, 561 (2018).
26. W. Zhou *et al.*, Efficiently controlling for case-control imbalance and sample relatedness in large-scale genetic association studies. *Nature Genetics* **50**, 1335-1341 (2018).
